# Neuromodulation Treatments of Pathological Anxiety in Anxiety Disorders, Stressor-Related Disorders, and Major Depressive Disorder: A Dimensional Systematic Review and Meta-Analysis

**DOI:** 10.3389/fpsyt.2022.910897

**Published:** 2022-07-01

**Authors:** Florian Gay, Allison Singier, Bruno Aouizerate, Francesco Salvo, Thomas C. M. Bienvenu

**Affiliations:** ^1^Université de Bordeaux, Bordeaux, France; ^2^Centre de Référence Régional des Pathologies Anxieuses et de la Dépression, Pôle de Psychiatrie Générale et Universitaire, Centre Hospitalier Charles Perrens, Bordeaux, France; ^3^Bordeaux Population Health, Inserm U1219, Bordeaux, France; ^4^NutriNeuro, UMR 1286, INRAE, Bordeaux INP, Bordeaux, France; ^5^CHU de Bordeaux, Bordeaux, France; ^6^Neurocentre Magendie, Inserm U1215, Bordeaux, France

**Keywords:** DBS (deep brain stimulation), tDCS – transcranial direct current stimulation, rTMS (repetitive transcranial magnetic stimulation), anxiety, meta-analysis

## Abstract

**Background:**

Pathological anxiety is responsible for major functional impairments and resistance to conventional treatments in anxiety disorders (ADs), posttraumatic stress disorder (PTSD) and major depressive disorder (MDD). Focal neuromodulation therapies such as transcranial magnetic stimulation (TMS), transcranial direct current stimulation (tDCS) and deep brain stimulation (DBS) are being developed to treat those disorders.

**Methods:**

We performed a dimensional systematic review and meta-analysis to assess the evidence of the efficacy of TMS, tDCS and DBS in reducing anxiety symptoms across ADs, PTSD and MDD. Reports were identified through systematic searches in PubMed/Medline, Scopus and Cochrane library (inception to November 2020), followed by review according to the PRISMA guidelines. Controlled clinical trials examining the effectiveness of brain stimulation techniques on generic anxiety symptoms in patients with ADs, PTSD or MDD were selected.

**Results:**

Nineteen studies (RCTs) met inclusion criteria, which included 589 participants. Overall, focal brain activity modulation interventions were associated with greater reduction of anxiety levels than controls [SMD: −0.56 (95% CI, −0.93 to−0.20, *I*^2^ = 77%]. Subgroup analyses revealed positive effects for TMS across disorders, and of focal neuromodulation in generalized anxiety disorder and PTSD. Rates of clinical responses and remission were higher in the active conditions. However, the risk of bias was high in most studies.

**Conclusions:**

There is moderate quality evidence for the efficacy of neuromodulation in treating pathological anxiety.

**Systematic Review Registration:**

https://www.crd.york.ac.uk/prospero/display_record.php?RecordID=233084, identifier: PROSPERO CRD42021233084. It was submitted on January 29th, 2021, and registered on March 1st, 2021. No amendment was made to the recorded protocol. A change was applied for the subgroup analyses based on target brain regions, we added the putative nature (excitatory/inhibitory) of brain activity modulation.

## Introduction

Pathological anxiety is a hallmark of mental disorders encompassing anxiety disorders, posttraumatic stress disorder and major depressive disorder. Anxiety disorders (ADs), including generalized anxiety disorder (GAD), panic disorder (PD), social anxiety disorder (SAD) and specific phobias (SP), are characterized by a functional impairment due to excessive anxiety. Anxiety is also a core symptom cluster in posttraumatic stress disorder [(PTSD; (1)]. Anxiety is a possible symptom in major depression, and anxious forms of depressive disorders are characterized by higher clinical severity (2–4). Moreover, ADs, PTSD and major depressive disorder (MDD) are highly comorbid internalizing disorders (5). They affect a large population, with lifetime prevalence of 15–20%, 2–7%, and 13–16%, respectively (6, 7), and cause heavy individual and financial burden, summing up to over 150 billion €/year in Europe (8–11). Current therapeutic strategies to manage these conditions rely on pharmacology (antidepressants) and psychotherapy (chiefly cognitive based therapy). However, more than one third of patients with ADs, PTSD or MDD are unresponsive to those conventional treatments (12–15). The intensity of anxiety in MDD is itself predictive of poor therapeutic outcomes (16, 17). As a result, effective treatment of pathological anxiety remains an unmet, pressing need. Key brain regions implicated in ADs, PTSD and MDD are largely overlapping, including the prefrontal cortex and the amygdala (18–21), suggesting that interventions aiming at restoring physiological functioning in this brain network may represent complementary therapeutic options.

In line with this, heavy research efforts are being invested in the development of novel therapeutic tools to control brain activity in ADs, PTSD and MDD, including neuromodulation techniques. Available neuromodulation techniques modify the electrical activity of targeted brain areas with the application of external magnetic (Transcranial Magnetic Stimulation – TMS) or electrical fields (transcranial Direct Current Stimulation- tDCS), or direct intracerebral currents (Deep Brain Stimulation- DBS) (22–24). In TMS, magnetic pulses induce action potentials in local neuronal populations, and their repetition at low or high frequency inhibits or facilitates synaptic transmission and neuronal firing (23). In tDCS, the locally induced electrical field alters membrane polarization and excitability at subthreshold levels, resulting in neuronal firing facilitation dependent on ongoing synaptic activities in areas close to the anode, and inhibition near the cathode (24). The mechanisms of action of DBS remain poorly understood, potentially involving depolarization of axonal fibers, excitation of local neurons (principal neurons and interneurons), or modulation of firing-patterns of neuronal populations (22). In MDD, TMS and tDCS have demonstrated clinical efficacy (25–27) and DBS has shown promising results (28, 29). ADs and PTSD may benefit from TMS and tDCS, as summarized in several recent reviews (30–34) and meta-analyses of the literature (35–37). These neuromodulatory techniques might also provide beneficial effects on MDD-related anxiety. In keeping with their clinical similarities and shared neurobiological substrates, brain stimulation protocols in ADs, PTSD and MDD are similar, with the most frequent procedures consisting in the activation of the left and/or inhibition of the right dorsolateral prefrontal cortex (dlPFC) (26, 27, 34, 35).

Paradoxically, while ADs, PTSD and MDD are neurobiologically related and neurostimulation strategies applied in these disorders are similar, recent quantitative syntheses of the literature have assessed their efficacy on the basis of disorder-specific psychometric measurements (35–37). This categorial approach evaluates symptoms that belong to cognitive, affective, or behavioral domains, with items differing among disorders. Such heterogeneity precludes the global evaluation, and the synthesis of knowledge in neuroscience-based brain stimulation for pathological anxiety. In contrast, a dimensional, transdiagnostic evaluation is critical to evaluate and improve neuroscience-based treatments of pathological anxiety.

To tackle this limitation in the literature, we applied a dimensional approach to evaluate brain stimulation-based treatments for pathological anxiety across related disorders. Systematic review and meta-analysis were performed with the main objective to study the efficacy of spatially targeted brain stimulation techniques on the intensity of generic anxiety symptoms in ADs, PTSD and MDD. The secondary objective was to synthetize available evidence for anxiety reduction with a categorial approach for ADs and PTSD, based on disorder-specific rating scales. This will facilitate comparisons with previous, categorial studies, and will update available evidence.

## Methods

### Search Strategy

This systematic review was conducted following the PRISMA guidelines (38). Two independent investigators performed the review (FG, TB). A literature search was performed on Pubmed, Scopus, and the Cochrane library, from inception until 31 November 2020. The following terms were used on the three databases: (“brain stimulation” OR “TMS” OR “RTMS” OR “Transcranial Magnetic Stimulation” OR “Theta burst” OR “transcranial direct current stimulation” OR “TDCS” OR “DBS” OR “neuromodulation”) AND (“Anxiety disorders” OR “agoraphobia” OR “social, Anxiety” OR “phobic disorders” OR “Panic disorder” OR “Phobia” OR “stress disorder, post-traumatic” OR “PTSD” OR “anxious symptoms” OR “HAMA” OR “Hamilton anxiety” OR “Anxiety scale” OR “Beck Anxiety Inventory”). Unpublished studies were sought with Google Scholar. After excluding duplicates (both automatically with Excel and manually), titles and abstracts were screened to select potentially eligible articles, which were then studied in their complete forms. Additional articles were identified in previously published reviews and in reference lists of selected studies. Only original articles written in English or French languages were considered eligible for inclusion. Each retrieved record was assessed by two investigators (FG, TB).

### Inclusion and Exclusion Criteria

Clinical trials examining the effectiveness of brain stimulation techniques on anxiety symptoms in patients with ADs, PTSD, or MDD were selected in accordance with PICOS (Participants, Intervention, Comparison, Outcomes and Study design) inclusion criteria: (1) clinical trials including adult patients with GAD, PD, PTSD, SP, SAD, or MDD, diagnosed according to DSM-IV, DSM-5 or ICD-10 criteria, without restriction regarding age, concomitant treatment, or date of symptom onset; (2) therapeutic intervention with TMS, tDCS, or DBS, (3) outcome measurement with at least one validated quantitative generic rating scale of anxiety symptoms including the Hamilton Anxiety Rating Scale (HARS), the Beck Anxiety Inventory (BAI), the Hamilton Depression Rating Scale (HAMD-17) anxiety-somatization factor items, or the State-Trait Anxiety Inventory (STAI), (4) controlled designs, both parallel groups or crossover, with or without randomization or blinding.

Studies on patients suffering from bipolar disorders, obsessive-compulsive disorder, schizophrenia, organic psychiatric disorders (DSM-5), or neurological conditions, and pregnant subjects, were not included. Articles corresponding to other stimulation techniques were not included, namely: Alpha-Stim Cranial Electrotherapy Stimulation (CES), Low-Charge Electrotherapy (LCE), rhythmic Low-Field Magnetic Stimulation (LFMS), Magnetic Resonance therapy (MRT), Radio Electric Asymmetric Conveyor brain stimulation (REAC), Transcranial Doppler ultrasound (TCD), Trigeminal Nerve Stimulation (TNS), Transcranial Photobiomodulation (t-PBM), transcranial Random Noise Stimulation (tRNS), and transcutaneous Vagus Nerve Stimulation (tVNS). Articles reporting initial results were not included when data were duplicated in follow-up studies. Excluded articles types were review articles, articles which did not contain original data (protocols, editorials, commentaries, notes), retrospective studies, case reports or series, animal or preclinical studies, and studies on healthy volunteers. Any disagreement in article selection was resolved by discussion between the authors, until a consensus was reached.

### Data Extraction

Included articles were reviewed to extract relevant data : article indexing information (authors, year of publication), study design (control, randomization, blinding), sample characteristics (diagnosis, number of patients in each arm of treatment, mean age, gender ratio), brain stimulation protocols (technique, target brain region, target identification method, stimulation and sham systems descriptions, stimulation settings, duration and number of sessions), concomitant therapeutic interventions, symptom measurement tools, dropout rates, and pre- and post-treatment data, including follow-up data (attrition rates, mean score and standard deviation of scale ratings, response and remission definitions and rates, adverse events). Study authors were contacted directly to retrieve data unavailable in original publications (twice in the absence of response to the first request). Data were collected in the form of an electronic spreadsheet (Excel).

### Outcomes

The primary outcome was the difference in intensity of anxiety symptoms between the baseline and the end of the treatment period (pre- and post-treatment), measured using validated, generic, quantitative scales of anxiety. Pre-specified secondary outcomes were: (1) the rate of clinically significant response or remission (as defined by study authors), (2) the intensity of anxiety symptoms at the last available follow-up (using the same scales as in the primary outcome analysis), (3) the difference in the severity of stressor-related and anxiety disorders between baseline and the end of the treatment period measured using validated, disorder-specific quantitative scales, (4) the adverse events rates and nature.

### Strategy for Data Synthesis (Meta-Analysis)

Analysis was performed using R software (R® 4.1.2). For the treatment and control groups of each study, we extracted (1) baseline/post-treatment mean differences in general anxiety rating scales and/or specific disorder rating scales, and (2) response and remission rates. The change-from-baseline SD was calculated following guidelines of the Cochrane Handbook for Systematic Reviews of Interventions (39).


$$\begin{array}{l} SD_{change}=\sqrt{{SD_{pre}}^{2}+{SD_{post}}^{2}-2\times Corr\times SD_{pre}\times SD_{post}} \end{array}$$

Where Corr represent the correlation between pre- and post-treatment variance, and was set to 0.5 as suggested by Follman et al. (40).

We calculated between-group standardized mean difference (SMD) and their variance for continuous variables, and Hedges' g to control for small sample bias, as suggested by Harrer et al. (41). Relative Risk and their variance were computed using the “escalc” function of the “epitools” package. The meta-analysis was conducted using a random model with the “rma.mv” function of the “metafor” package. A multi-level model was fitted to account for multiple active or control groups and to control for dependencies between effect sizes. Comparisons were made between active brain stimulation and control conditions. When two validated quantitative scales evaluating the same outcome were available (such as HARS and BAI), results from the scale most frequently found in the other studies were selected for the meta-analysis (see Supplementary Material for details).

Pre-specified subgroup analyses of primary and secondary outcomes were performed for specific disorders (GAD, MDD, PD, PTSD, SAD, SP), stimulation techniques (tDCS, TMS, DBS) and target brain regions. From the pre-registered protocol, we modified the latter subgroup definition by categorizing the putative nature (excitatory/inhibitory) of brain activity modulation. Neuromodulation was considered excitatory for TMS for frequencies ≥ 5 Hz, intermittent theta burst stimulation (iTBS) and for brain regions located below anodes in tDCS. Stimulation was considered inhibitory for low TMS frequencies (mainly 1 Hz) and cathodal tDCS. Multisite neuromodulation protocols were not included in the analysis of discrete target regions. Likewise, when studies were composed of three arms, of which two were active conditions targeting distinct brain regions, individual active arms were included in the corresponding brain target subgroup.

Heterogeneity between studies was assessed by the Cochran Q test (with *p*-value < 0.10 considered statistically significant) and quantified by indicator *I*^2^ considered low if *I*^2^ ≤ 25%; moderate if 25% < *I*^2^ < 50%, and high if *I*^2^ > 50%.

A meta-regression analysis was conducted to explore potential sources of heterogeneity, using the “rma.mv” package. We assessed the putative moderation effect of the number of sessions of stimulation, the total number of pulses and the motor threshold percentage in TMS studies, the difference in anxiety scores at baseline between active and sham groups (SMD) and the difference between active and sham groups for baseline to post-treatment changes in depression and PTSD scores (SMD). The model was assessed for each factor one by one.

### Risk of Bias and Quality Assessment

Publication bias was investigated visually with funnel plots and statistically by applying the Egger's test (with *p*-value < 0.05 considered statistically significant; R 4.0.4, Metafor package). For each study, risk of bias was assessed using the Risk of Bias tool of the *Cochrane Collaboration* (39). Risk of bias was assessed by domains for each study and graded as low, uncertain or high. The global risk of bias for each study was estimated as follow: studies assessed with high risk in one domain or intermediate risk in more than three domains were considered as showing a high risk of bias. Studies with a maximum of two intermediate risk items were assigned to low risk of bias. Remaining studies were classified as showing an intermediate risk of bias. Quality of evidence across studies was determined using the GRADE approach (39).

## Results

### Literature Search Results and Characteristics of Included Studies

**Figure 1:**
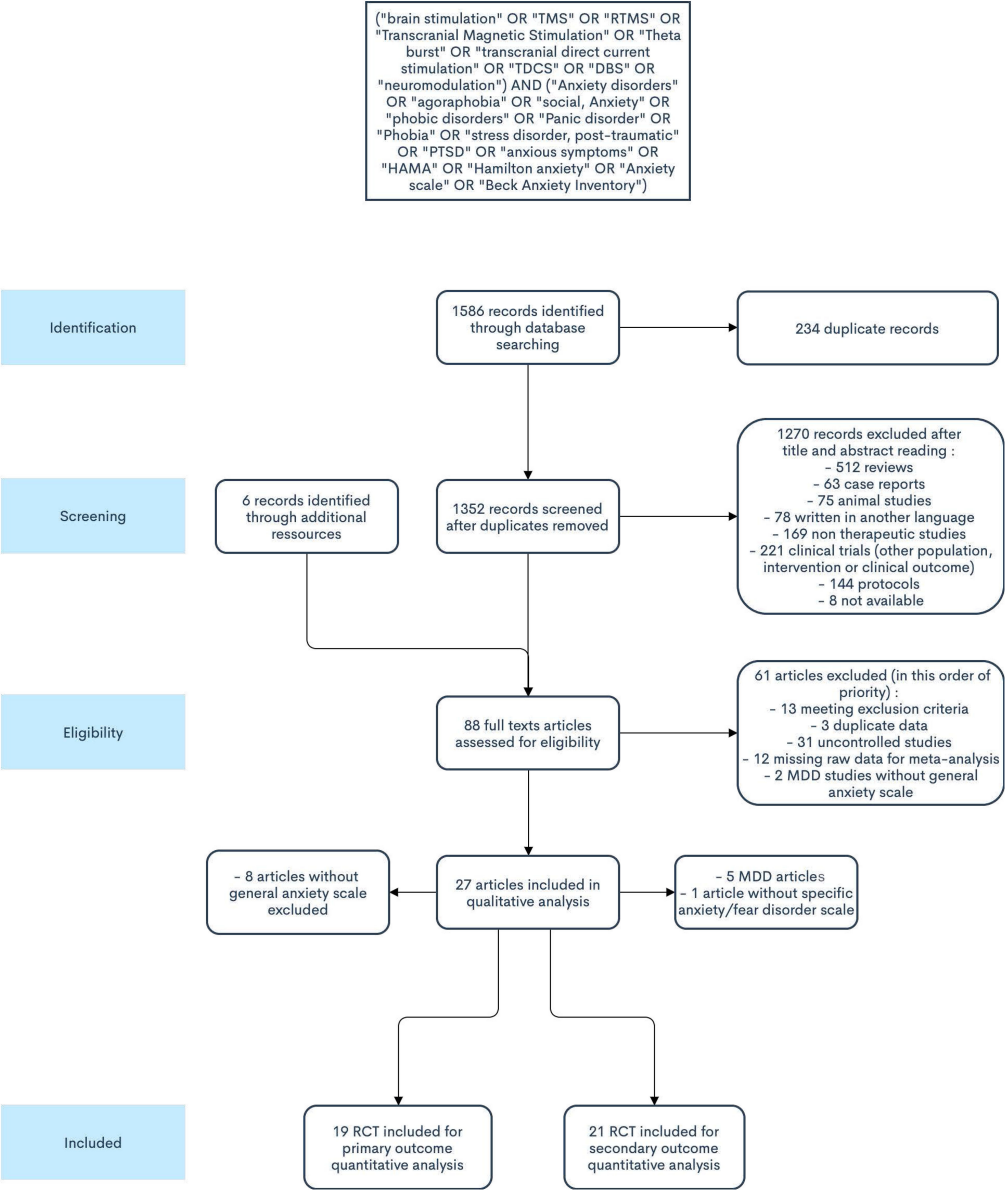
PRISMA flowchart.

A total of 1,586 records were identified through database search (Medline: 588, Scopus: 559, Cochrane: 439). Among those, 234 were duplicate references. Eighty-two articles were retrieved for inclusion assessment. After full-text reading and adding articles found through cross-referencing, 27 studies were included. All were randomized controlled trials (RCTs) (Figure 1). From these, 19 studies fulfilled all eligibility criteria for the primary outcome (42–60) (Tables 1, 2), for a total of 589 participants. The mean age of participants was 40.5 years, and 58.1% were females. Twelve trials assessed TMS interventions (three in GAD, three in PD, one in SP, three in PTSD, two in MDD), five trials tDCS interventions (three in GAD, one in PTSD, one in MDD), and two trials DBS interventions (two in MDD).

**Table 1:** Characteristics of included studies (diagnosis, study design, target, stimulation and sham protocols).

**Outcome in meta-analysis**	**Technique**	**References**	**Diagnosis**	**Study design**	**Target**	**Target identification**	**Pulses (TMS) or duration of stim (DCS/DBS) [n sessions]**	**Stimulation device**	**Sham procedure**
Primary outcome: anxiety symptoms intensity	TMS	Diefenbach et al. (47) (PO + SO)	GAD	Single center RCT, 1 Hz Right dlPFC rTMS (group A, *n* = 13) vs. Sham (group C, *n* = 12)	Right dlPFC 90%MT 1 Hz	MNI coordinates (*x* = 42, *y* = 36, *z* = 32) neuronavigation	900/session, 27,000 total [30]	NR	Sham coil
		Dilkov et al. (48) (PO)	GAD	Two-center RCT, 20 Hz Right dlPFC rTMS (group A, *n* = 15) vs. Sham (group C, *n* = 25)	Right dlPFC 110% MT 20 Hz	5 cm rule (distance from the motor strip location that caused hand movement)	3,600/session, 90,000 total [25]	8-shape coil	90° angle between coil and scalp, same intensity
		Huang et al. (51) (PO + SO)	GAD (+ insomnia)	Single center RCT, 1 Hz Right PPC rTMS (group A, *n* = 18) vs. Sham (group C, *n* = 18)	Right PPC 90% MT 1 Hz	P4	1,500/session, 15,000 total [10]	8-shape coil	8-shape sham coil (looks, sounds, feels like active coil)
		Anderson et al. (43) (PO)	MDD	Single center RCT, 10 Hz Left dlPFC rTMS (group A, *n* = 13) vs. Sham (group C, *n* = 16)	Left dlPFC 110% MT 10 Hz	5 cm rule	1,000/session, 12,000 total (18,000 for partial responders) [12]	8-shape coil	Sham coil
		Triggs et al. (59) (PO)	MDD (TRD)	Single center RCT, 5 Hz Left dlPFC rTMS (group A, *n* = 18) vs. 5 Hz right dlPFC rTMS (group B, *n* = 16) vs. Sham right or left (group C, *n* = 14)	Right or Left dlPFC 100% MT 5 Hz	5 cm rule	2,000/session, 20,000 total [10]	8-shape coil	8-shape sham coil (looks and sounds like active coil)
		Deppermann et al. (46) (PO + SO)	PD	Two-center RCT, Left dlPFC iTBS (group A, *n* = 22) vs. Sham (group C, *n* = 22) as add-on for psychoeducation. Additional healthy controls (group B, *n* = 23)	Left dlPFC 80% MT iTBS	F3	600/session, 9,000 total [15]	8-shape coil	90° angle between coil and scalp
		Mantovani et al. (54) (PO + SO)	PD (+ TRD)	Single center RCT, 1 Hz Right dlPFC rTMS (group A, *n* = 12) vs. Sham (group C, *n* = 13)	Right dlPFC 110% MT 1 Hz	5 cm rule	1,800/session, 36,000 total [20]	8-shape coil	8-shape sham coil with mu-metal shield (looks and sounds but does not feel like active coil)
		Prasko et al. (56) (PO + SO)	PD (TR)	Single center RCT, 1 Hz Right dlPFC rTMS (group A, *n* = 7) vs. Sham (group C, *n* = 8) as add-on for SSRI	Right dlPFC 110% MT 1 Hz	5 cm rule	1,800/session, 18,000 total [10]	8-shape coil	90° angle between coil and scalp, same intensity
		Cohen et al. (44) (PO + SO)	PTSD	Single center RCT, 1 Hz (group A, *n* = 8) or 10 Hz Right dlPFC rTMS (group B, *n* = 10) vs. Sham (group C, *n* = 6)	Right dlPFC 80% MT 1 / 10 Hz	5 cm rule	100/session, 1,000 total (1 Hz); 400/session, 4,000 total (10 Hz) [10]	Circular coil	90° angle between coil and scalp, same intensity
		Leong et al. (52) (PO + SO)	PTSD	Single center RCT, 1 Hz (group A, *n* = 11) or 10 Hz Right dlPFC rTMS (group B, *n* = 9) vs. Sham (group C, *n* = 9)	Right dlPFC 120% MT 1 / 10 Hz	6 cm rule	2,250/session, 30,000 total (1 Hz)/3,000/session, 30,000 total (10 Hz) [10]	8-shape coil	Sham coil (looks, sounds and feels like active coil with vibratory somatosensory effect)
		Watts et al. (60) (PO + SO)	PTSD	Single center RCT, 1 Hz Right dlPFC rTMS (group A, *n* = 10) vs. Sham (group C, *n* = 10)	Right dlPFC 90% MT 1 Hz	4 cm anterior and 2 cm laterally of motor strip location	400/session, 4,000 total [10]	8-shape coil	8-shape sham coil (looks and sounds like active coil)
		Herrmann et al. (49) (PO + SO)	Specific phobia (acrophobia)	Single RCT, 10 Hz mPFC rTMS (group A, *n* = 20) vs. Sham (group C, *n* = 19), as add on to 2 virtual-reality exposure sessions.	mPFC 100% MT 10 Hz	FPz	1,560/session, 3,120 total [2]	circular coil	Sham circular coil (looks and sounds like active coil)
	tDCS	de Lima et al. (53) (PO + SO)	GAD	Two-center RCT, tDCS 2 mA (cathode Right supraorbital cortex, anode Left dlPFC) (group A, *n* = 15) vs. Sham (group C, *n* = 15)	Left dlPFC (anode), Right supraorbital area (cathode)2 mA	Fp2 (cathode), F3 (anode)	20 min [5]		Current ramped down after 30s
		Movahed et al. (58) (PO + SO)	GAD	Single center RCT, 2 mA tDCS (group A, *n* = 6) vs. pharmacotherapy (group B, *n* = 6) vs. Sham (group C, *n* = 6)	Right dlPFC (cathode) and controlateral deltoid (anode) 2 mA	F4 (cathode), controlateral deltoid (anode)	20 min [10]		NR
		Nasiri et al. (55) (PO + SO)	GAD (+ MDD)	Single center RCT, tDCS + UP (group A, *n* = 13) vs. UP alone (group B, *n* = 15) vs. waiting list as control (group C, *n* = 15)	Right dlPFC (cathode) and controlateral deltoid (anode) 2 mA	NR (cathode), controlateral deltoid (anode)	30 min [10]		No sham arm
		Dastjerdi et al. (45) (PO)	MDD	Single center RCT, Active tDCS + Active CES (group 1, *n* = 10) vs. Active tDCS + Sham CES (group B, *n* = 10) vs. Sham tDCS + Active CES (group C, *n* = 10)	Placement based on EEG patterns <2 mA	NR	20 min [6]		No sham arm
		Ahmadizadeh et al. (42)	PTSD	Single center RCT, tDCS 2 mA (cathode Right dlPFC, anode Left dlPFC) vs. Sham	Right (cathode) and Left (anode) dlPFC 2 mA	F4 (cathode), F3 (anode)	20 min [10]		Current ramped down after 30 s
	DBS	Holtzheimer et al. (50) (PO)	MDD (TRD)	Multicenter (20) RCT, bilateral subcallosal cingulate active DBS (group A, *n* = 56) vs. Sham (group C, *n* = 29)	Bilateral subcallosal cingulate 4 mA (progressively increased to 6/ 8 mA if partial response), 130 Hz, 91 μs pulse width,	Preoperative high-resolution MRI. Neurosurgeon trained on targeting by a team of experts. Control with postoperative CT scan merged with preoperative MRI.	for 6 months	Four-electrode array (2 active contacts)	Sham programming session was done, but stimulation was not initiated
		Puigdemont et al. (57) (PO)	MDD (TRD)	Single center RCT, bilateral subcallosal cingulate gyrus DBS vs. Sham, cross-over design, *n* = 5, after DBS-obtained remission	Bilateral subcallosal cingulate gyrus 3.5–5 V, 130–125 Hz, 120–240 μs pulse width	Neuronavigation with fused CT and MRI scans: coordinates of white/gray matter transition for BA 25 area.	3 months (3 ON/3 OFF)	Four electrode array	NR
secondary outcome: disorder-specific scales	TMS	Ahmadizadeh et al. (61) (SO)	PTSD	Single center RCT, 20 Hz Right dlPFC rTMS (group A, *n* = 19) vs. 20 Hz Bilateral dlPFC rTMS (group B, *n* = 19) vs. Sham (group C, *n* = 20)	Right vs. bilateral dlPFC 100% MT 20 Hz	5 cm rule	2,400/session, 24,000 total [10]	8-shape coil	8-shape sham coil (looks and sounds like active coil, plastic shell)
		Isserles et al. (62) (SO)	PTSD	Single center RCT, Active 20 Hz mPFC dTMS + Active Exposure (group A, *n* = 9) vs. Active dTMS + Sham exposure (group B, *n* = 8) vs. Sham dTMS + active exposure (group C, *n* = 9)	mPFC 120% MT 20 Hz	Midline of PFC	1,680/session, 20,160 total [12]	H-coil	H-coil sham (looks, sounds and feels like active coil, rapid reduction of the field)
		Kozel et al. (63) (SO)	PTSD	Single center RCT, 1 Hz Right dlPFC rTMS (group A, *n* = 54) vs. Sham (group C, *n* = 49) as add-on for CPT	right dlPFC 110% MT 1 Hz	F4	1,800/session, 21,600 total [12]	8-shape coil	8-shape sham coil (looks, sounds, but do not feel like active coil)
		Osuch et al. (64) (SO)	PTSD	Single center RCT, 1 Hz Right dlPFC rTMS vs. Sham, cross-over design, as add on to exposure therapy	Right dlPFC 100% MT 1 Hz	5 cm rule	1,800/session, 36,000/condition total [20 of each condition]	8-shape coil	45° angle between coil and scalp
		Philip et al. (65) (SO)	PTSD	Single center RCT, right dlPFC iTBS (group A, *n* = 25) vs. Sham (group C, *n* = 25)	Right dlPFC 80% MT iTBS	F4	1,800/session, 18,000 total [10]	NR	Sham coil
		Philip et al. (65) (SO)	PTSD (+ MDD)	Two centers RCT, synchronized TMS on IAF (group A, *n* = 10) vs. Sham (group C, *n* = 13) (+ 4 weeks of optional unblinded sTMS)	NR	NR	[20]	NR	Sham coil
	tDCS	Smits et al. (66) (SO)	PTSD	Single center RCT, right IFG 1.25 mA tDCS (group A, *n* = 23) vs. Sham (group C, *n* = 23)	Right IFG 1.25 mA	Left orbital region (cathode), crossing point between T4-Fz and F8-Cz (anode)	20 min [5]		6-s fade-in fade-out stimulation at the start and end of the stimulation period, interleaved by occasional 15 ms pulses of 0.11 mA
		Van't Wout-Frank et al. (67) (SO)	PTSD	Single center RCT, 2 mA on vmPFC tDCS (group A, *n* = 6/12) vs. Sham (group C, *n* = 6/12)	vmPFC 2 mA	PO8 (cathode), AF3 (anode)	25 min [6]		NR

***Intervention:** CES, cranial electrical stimulation; CPT, cognitive processing therapy; DBS, deep brain stimulation; EEG, electroencephalogram; iTBS, intermittent theta burst stimulation; SSRI, serotonin selective reuptake inhibitor; tDCS, transcranial direct current stimulation; TMS, transcranial magnetic stimulation (s-synchronized, r-repetitive, d-deep); UP, Unified Protocol for Transdiagnostic Treatment of Emotional Disorders.*

***Disorders:** GAD, generalized anxiety disorder; MDD, major depressive disorder; PD, panic disorder; PTSD, post-traumatic stress disorder; SP, specific phobia; TR, treatment-resistant; TRD, treatment-resistant depression.*

***Targets:** PFC, prefrontal cortex (vm-ventromedial; dl-dorsolateral; m-medial); PPC, posterior parietal cortex; IFG, inferior frontal gyrus; IAF, intrinsic alpha frequency; ITT, intent to treat; MNI, Montreal neurological institute; MT, motor threshold; NR, not reported; PP, per protocol; RCT, randomized controlled trial.*

***Psychometric scales:** AQ, Acrophobia Questionnaire; BAI, Beck anxiety inventory; BDI, Beck depression inventory; CAPS, clinician-administered PTSD scale; GAD-Q-IV, generalized anxiety disorder questionnaire IV; HAD, Hospital anxiety and depression scale; HARS, Hamilton anxiety rating scale; HDRS, Hamilton depression rating scale; ISSL, Lipp inventory of stress symptoms for adults; MADRS, Montgomery Asberg depression rating scale; PAS, panic and agoraphobia scale; PCL, PTSD checklist; PDSS, panic disorder severity scale; PSQI, Pittsburgh sleep quality index; PSWQ, Penn state worry questionnaire; QIDS, Quick inventory of depressive symptomatology; STAI, state trait anxiety inventory (STAI-S: state; STAI-T: trait); TOP-8, treatment outcome PTSD Scale.*

*PO, Primary outcome; SO, secondary outcome*.

**Table 2:** Characteristics of included studies (extracted scales, drop out, analysis, response and remission rates).

**Outcome in meta-analysis**	**Technique**	**References**	**Extracted scale(s) for meta-analysis (top:primary; bottom:secondary)**	**Drop out (*n*)**	**Analysis**	**Response**	**Remission**
Primary outcome: anxiety symptoms intensity	TMS	Diefenbach et al. (47)	HARS PSWQ	1	Modified ITT (PP but not reported)	≥50% HARS improvement: 8/13 (active) vs. 2/12 (sham), *p*=0,022	HARS <8 and CGI ≤ 2: 4/13 (active) vs. 1/12 (sham), ns
		Dilkov et al. (48)	HARS	10	PP	≥50% HARS improvement: 15/15 (active) vs. 3/25 (sham)	HARS <10: 12/15 (active). Sham NR.
		Huang et al. (51)	HARS PSQI	0	ITT	≥50% HARS improvement: 8/14 (active) vs. 0/18 (sham), *p* = 0.003	HARS <8: 5/18 (active) vs. 0/18 (sham), *p* = 0.045
		Anderson et al. (43)	HAD anxiety	4	PP	≥50% HARS improvement + CGI–I rating of much or very much improved: 6/14 (active) vs. 1/14 (sham), *p* < 0.05	–
		Triggs et al. (59)	STAI-S	uncertain: 13 withdrew consent	Unknown	≥50% HARS improvement: 6/18 (active left) vs. 8/16 (active right) vs. 4/14 (sham)	–
		Deppermann et al. (46)	HARS PAS	NR	Unknown	–	–
		Mantovani et al. (54)	HARS PDSS	4	ITT	≥40% decrease of PDSS: 6/12 (active) vs. 1/13 (sham)	PDSS <5: 3/12 (active) vs. 0/13 (sham)
		Prasko et al. (56)	HARS PDSS	0	ITT	–	–
		Cohen et al. (44)	HARS PCL	5	PP	–	–
		Leong et al. (52)	BAI PCL	3	Modified ITT + PP	–	–
		Watts et al. (60)	STAI PCL	0	ITT	–	–
		Hermann et al. (49)	AQ-anxiety	8	PP	–	–
	tDCS	de Lima et al. (53)	HARS ISSL	0	ITT	–	–
		Movahed et al. (58)	HARS PSWQ	NR	Unknown	–	–
		Nasiri et al. (55)	BAI PSWQ	4	PP	–	Diagnostic criteria for GAD (DSM-5) no longer met: 9/13 (tDCS + UP) vs. 9/15 (UP alone) vs. 0/15 (waiting list)
		Dastjerdi et al. (45)	BAI	NR	Unknown	–	–
		Ahmadizadeh et al. (42)	BAI PCL	6	PP	PCL-5 <33: 8/18 (active), 2/16 (sham), *p* = 0.046	Not remission but meaningful clinical improvement: PCL-5 ≤ 24 and reliable change index > 1.96. 5/18 (active) vs. 1/16 (sham), ns
	DBS	Holtzheimer et al. (50)	HARS	5	PP	≥50% improvement MADRS score averaged months 4–6: 7/56 (active), 3/29 (sham), ns	MADRS <10 score averaged months 4–6 months: 3/56 (active) vs. 2/29 (sham), ns
		Puigdemont et al. (57)	HDRS (anxiety items)	0	ITT	–	HAMD <8: 4/5 (active) vs. 2/5 (sham)
Secondary outcome: disorder-specific scales	TMS	Ahmadizadeh et al. (61)	PCL-M	7	Modified ITT	Improvement of ≥ 2 SD relative to the baseline total PCL score: 7/17 (right) vs. 10/16 (bilateral) vs. 0/17 (sham). *p*(right vs. sham) = 0.004, *p*(bilat vs. sham) = 0.0001	–
		Isserles et al. (62)	CAPS	5	PP	>50% improvement in CAPS score: 4/9 (active + exposure therapy) vs. 1/8 (active alone) vs. 0/9 (sham)	–
		Kozel et al. (63)	PCL	44	Modified ITT	–	–
		Osuch et al. (64)	CAPS	1	PP	–	–
		Philip et al. (68)	PCL	3	ITT	–	–
		Philip et al. (65)	PCL	1	ITT	–	–
		Smits et al. (66)	PCL	4	PP	–	–
		Van't Wout-Frank et al. (67)	PCL	NR	Unknown	–	–

*Footnote same as Table 1*.

Twenty-one RCTs were included for the secondary analysis, which was based on disorder-specific scales. Those 21 studies comprised 13 RCTs included in the primary outcome analysis [exclusion of one trial without specific scale measure (48) and five MDD trials (43, 45, 50, 57, 59)], and eight additional RCTs, in which only disorder-specific scores were reported (61–68) (Tables 1, 2). This secondary analysis involved 712 subjects (mean age: 40.3 years; females: 49.9%). Of the 21 studies, 15 trials assessed TMS interventions (two in GAD, three in PD, nine in PTSD, one in SP), and six assessed tDCS interventions (three on GAD symptoms, three on PTSD symptoms). No controlled trial using DBS was found for anxiety or stressor-related disorders. Although not excluded a priori, no article reporting continuous Theta Burst Stimulation (cTBS) protocol was found. Detailed reporting of patients' characteristics, study designs and neurostimulation protocols are provided in the Supplementary Material.

### Primary Outcome: Change in Anxiety Symptom Intensity

#### Main Result

**Figure 2:**
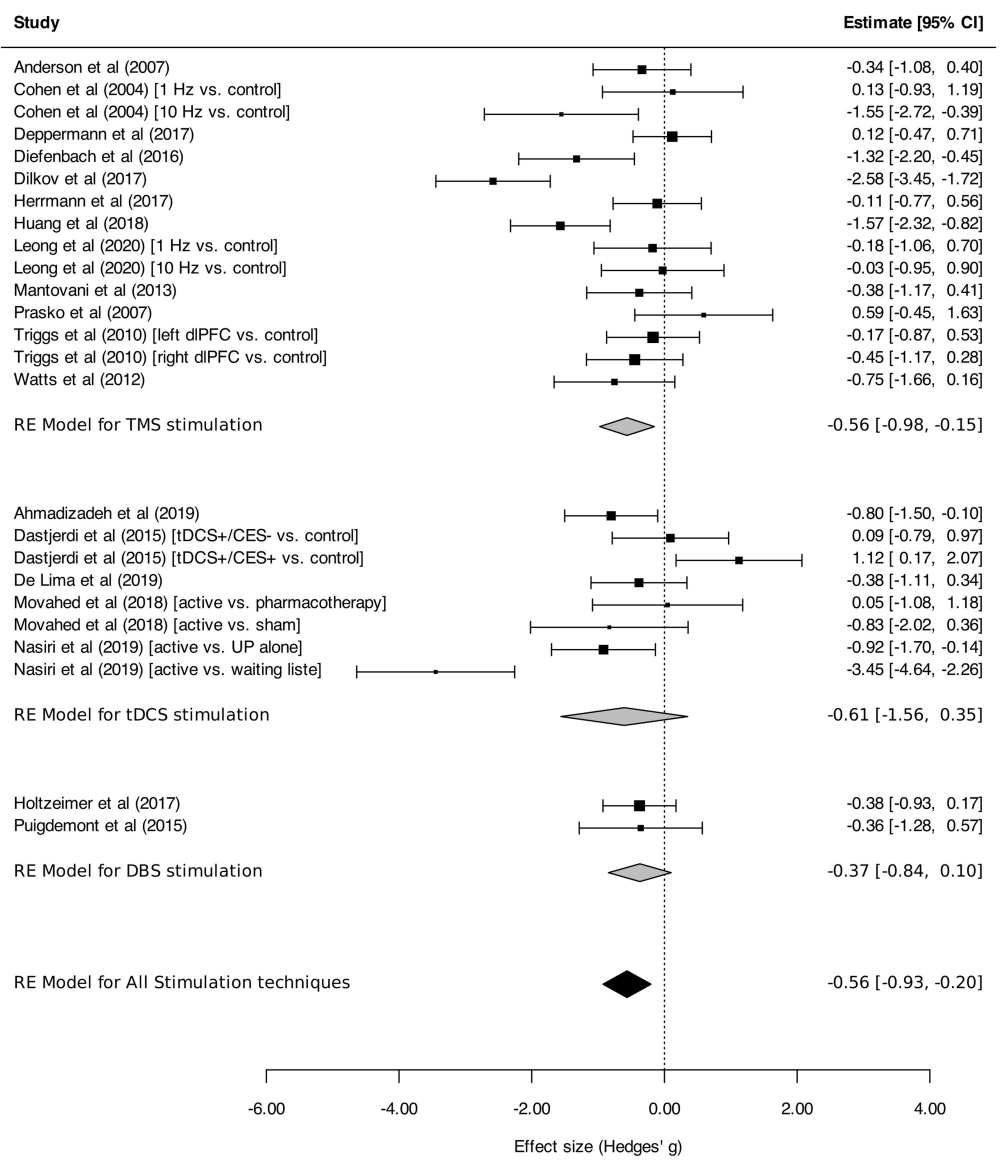
Change in the anxiety symptoms intensity in anxiety, stressor-related disorders and MDD, subgroups by stimulation technique. Forest plot displaying effect estimates and confidence intervals for both individual studies and overall meta-analyses. Each study is represented by a block at the point estimate of the intervention SMD, with a horizontal line extending on each side representing the confidence interval. The area of the block is proportional to the weight assigned to that study in the meta-analysis. When comparisons were made between three arms, the corresponding conditions, active or control (preceded with “vs”), are indicated between brackets. Statistics: TMS: heterogeneity Sigma1^2^ = 0.00, Sigma2^2^ = 0.48; Q(df = 14) = 50.8 (*p* < 0.0001); *I*^2^ = 74%; test for overall effect: *Z* = −2.68 (*p* = 0.007); tDCS: heterogeneity Sigma1^2^ = 0.53, Sigma2^2^ = 0.79; Q(df = 7) = 39.6 (*p* < 0.0001); *I*^2^ = 86%; test for overall effect: *Z* = −1.24 (*p* = 0.21); DBS: heterogeneity Sigma1^2^ = 0.00, Sigma2^2^ = 0.00; Q(df = 1) = 0.001 (*p* < 0.97); *I*^2^ = 0%; test for overall effect: *Z* = −1.54 (*p* = 0.12); Total: heterogeneity Sigma1^2^ = 0.20, Sigma2^2^ = 0.37; Q(df = 24) = 90.8 (*p* < 0.0001); *I*^2^ = 77%; test for overall effect: *Z* = −3.0599 (*p* = 0.002); test for subgroup differences: F(df1 = 2, df2 = 22) = 0.05, *p* = 0.95.

Neurostimulation was associated with a statistically significant reduction in the intensity of anxiety symptoms across ADs, PTSD and MDD [overall SMD, −0.56 (95% CI, −0.93 to −0.20), *I*^2^ = 77%], with a medium effect size (SMD > 0.5; Figure 2).

#### Subgroup Analyses

**Figure 3:**
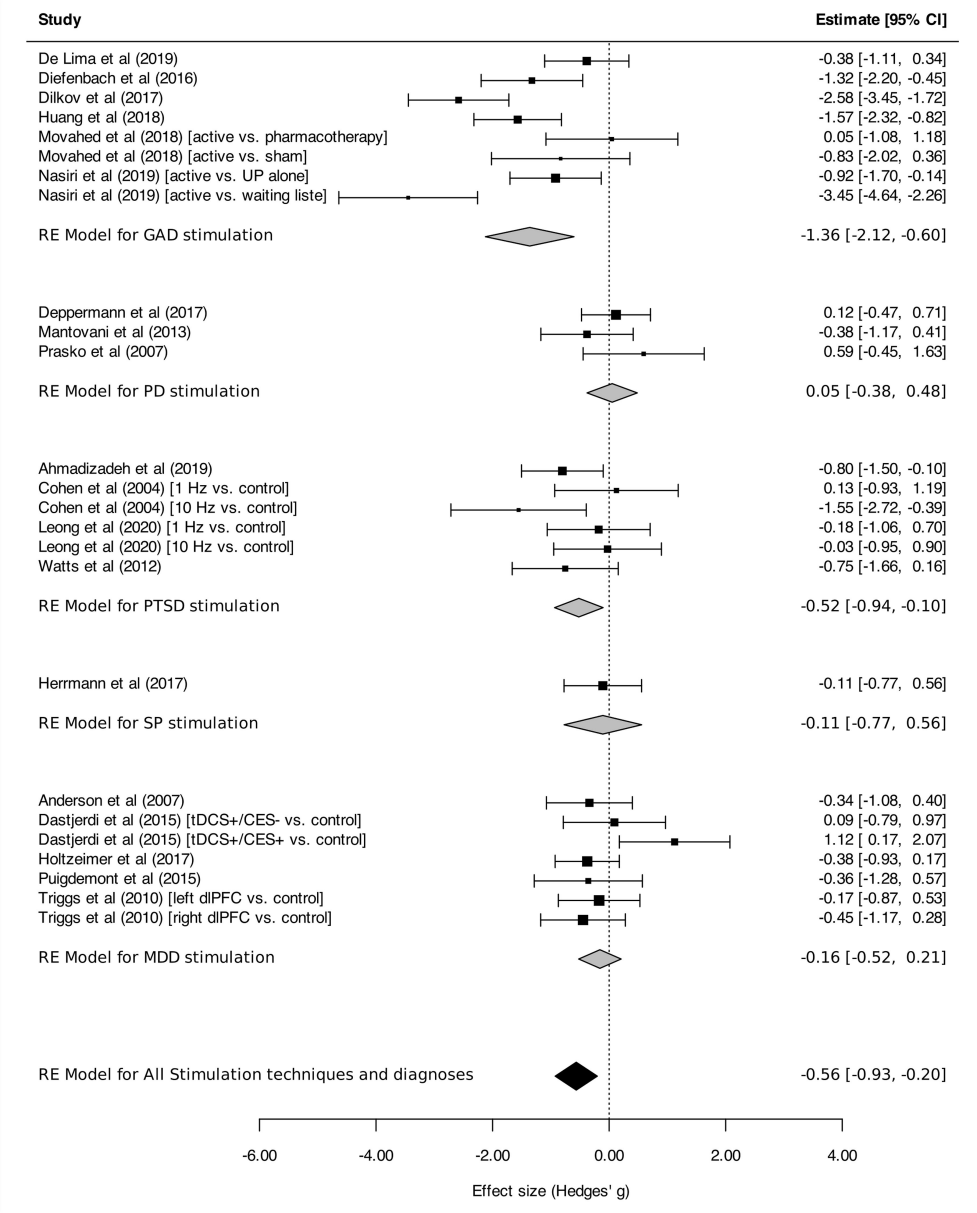
Change in the anxiety symptoms intensity in anxiety, stressor-related disorders and MDD, subgroups by disorder. Statistics: GAD: heterogeneity Sigma1^2^ = 0.00, Sigma2^2^ = 0.97; Q(df = 7) = 34.64, *p* < 0.0001); *I*^2^ = 82%; test for overall effect: *Z* = −3.52 (*p* = 0.0004); PD: heterogeneity Sigma1^2^ = 0.00, Sigma2^2^ = 0.00; Q(df = 2) = 2.23, *p* = 0.33); *I*^2^ = 00%; test for overall effect: *Z* = 0.24 (*p* = 0.81); PTSD: heterogeneity Sigma1^2^ = 0.00, Sigma2^2^ = 0.05; Q(df = 5) = 7.01, *p* = 0.22); *I*^2^ = 20%; test for overall effect: *Z* = −2.44 (*p* = 0.22); SP: heterogeneity : not applicable; test for overall effect: *Z* = −0.32 (*p* = 0.75); MDD: heterogeneity Sigma1^2^ = 0.00, Sigma2^2^ = 0.05; Q(df = 5) = 7.01, *p* = 0.22); *I*^2^ = 20%; test for overall effect: *Z* = −2.44 (*p* = 0.22); Total: heterogeneity Sigma1^2^ = 0.06, Sigma2^2^ = 0.00; Q(df = 6) = 8.94, *p*-val = 0.18); *I*^2^ = 30%; test for overall effect: *Z* = −3.0599 (*p* = 0.002); test for subgroup differences: F(df1 = 4, df2 = 20) = 3.69, *p* = 0.02.

**Figure 4:**
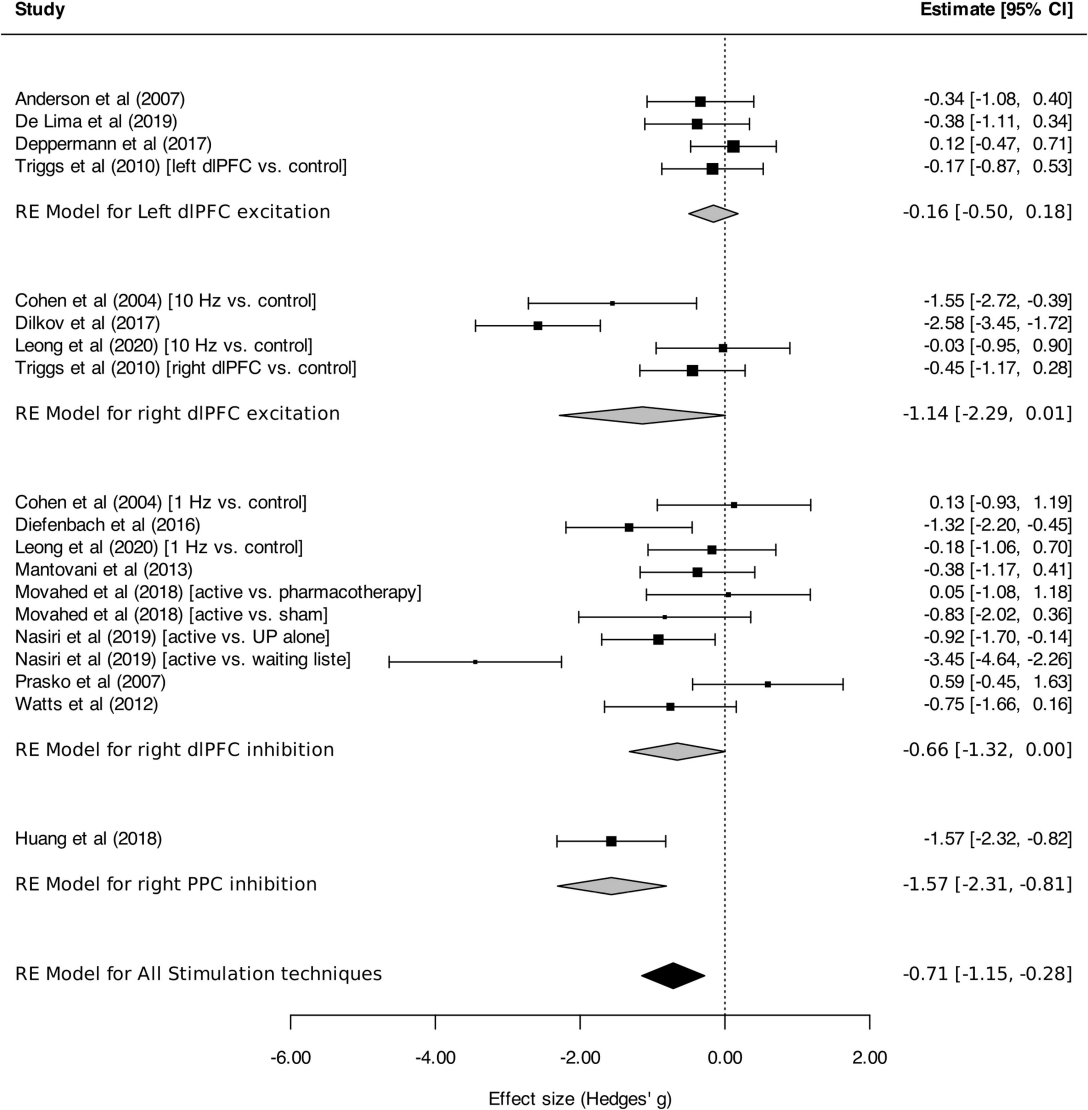
Change in the anxiety symptoms intensity in anxiety, stressor-related disorders and MDD, subgroups by target brain region. Statistics: left dlPFC excitation: heterogeneity Sigma1^2^ = 0.00, Sigma2^2^ = 0.00; Q(df = 3) = 1.44, *p* = 0.70; *I*^2^ = 00%; test for overall effect: *Z* = −0.91 (*p* = 0.36); right dlPFC excitation: heterogeneity Sigma1^2^ = 0.57, Sigma2^2^ = 0.57; Q(df = 3) = 20.21, *p* = 0.0002; test for overall effect: *Z* = −1.95 (*p* = 0.0002; *I*^2^ = 85%); right dlPFC inhibition: heterogeneity Sigma1^2^ = 0.15, Sigma2^2^ = 0.68; Q(df = 9) = 34.66, *p* < 0.0001; *I*^2^ = 78%; test for overall effect: *Z* = −1.95 (*p* = 0.05); right PPC (posterior parietal cortex) inhibition: heterogeneity : not applicable; test for overall effect: *Z* = −4.09 (*p* < 0.0001).

Regarding stimulation techniques, results in the TMS subgroup showed moderate reduction in anxiety symptom severity [SMD_TMS_: −0.56 (95% CI, −0.98 to −0.15), *I*^2^ = 74%]. Similar results were obtained in tDCS and DBS subgroups, with a tendency toward decline of anxiety symptom intensity, although without reaching statistical significance [SMD_tDCS_ : −0.61 (95% CI, −1.56 to 0.35), *I*^2^ = 86%; SMD_DBS_: −0.37 (95% CI, −0.84 to 0.10), *I*^2^ = 0%; Figure 2]. Subgroup analyses performed by disorder showed effects on anxiety in favor of neurostimulation in GAD and PTSD [SMD_GAD_: −1.36 (95% CI, −2.12 to −0.60), *I*^2^ = 82%; SMD_PTSD_: −0.52 (95% CI, −0.94 to −0.10), *I*^2^ = 20%; Figure 3]. In contrast, no beneficial effect of neurostimulation was found in PD [SMD_PD_: 0.05 (95% CI, −0.38 to 0.48), *I*^2^ = 0%), in SP (SMD_SP_: −0.11 (95% CI, −0.77 to 0.56), or in MDD (SMD_MDD_: −0.16 (95% CI, −0.52 to 0.21), *I*^2^ = 30%]. As for target brain region subgroups, the vast majority of protocols targeted the dlPFC (left dlPFC excitation: four RCTs, right dlPFC excitation: four RCTs, right dlPFC inhibition: eight RCTs). Right dlPFC inhibition was significantly associated with a reduction of anxiety levels across disorders [SMD: −0.66 (95% CI, −1.32 to −0.00), *I*^2^ = 78%], as was right dlPFC excitation but without strictly reaching significance in this case [SMD: −1.14 (95% CI, −2.29 to 0.01), *I*^2^ = 85%]. No evidence for therapeutic benefit was found for left dlPFC excitation [SMD: −0.16 (95% CI, −0.50 to 0.18), *I*^2^ = 0%]. A single study targeted the right posterior parietal cortex (PPC), with positive results [SMD: −1.57 [95% CI, −2.31 to −0.81; (51)] (Figure 4). Other trials applied bilateral dlPFC (42), ventromedial PFC (49) or bilateral cingulate cortex (50, 57). The ventromedial PFC study was not included in this analysis because the side of the stimulation was not specified (49). Stimulation protocols and results are reported in Tables 3, 4.

**Table 3:** Characteristics of included studies (socio-demographic data, blinding protocols, primary and secondary outcomes, other treatments, adverse events).

**Outcome in meta-analysis**	**Technique**	**References**	**Demographics**		**Blinding**	**Primary outcome**	**Secondary outcome**	**Other treatments**		**Adverse events**
			**Age**	**Male %**				**Pharmacotherapy**	**Psychotherapy**	
Primary outcome: anxiety symptoms intensity	TMS	Diefenbach et al. (47)	44.00 ± 11.95 (active); 44.58 ± 14.75 (sham)	24.0%	Subjects, raters, and administrators blind	Anxiety level (HARS)	PSWQ, DASS-DEP, HDRS-17	Allowed if dose stable for ≥3 months (except for benzodiazepine, stable ≥2 weeks)	No. Exclusion	1 chest pain unrelated to intervention. Most frequent: pain at stimulation point (*n* = 19) and pin prick sensation (*n* = 19), then headache (*n* = 9), facial twitch (*n* = 6), facial pain (*n* = 4), toothache (*n* = 3), dizziness (*n* = 2). Only facial twitch significantly more frequent in active group.
		Dilkov et al. (48)	34 ± 7 (active); 38 ± 10 (sham)	52.5%	Subjects (*) and raters blind. Administrators not blind.	Anxiety level (HARS)	CGI, HDRS-21	Allowed if dose stable for ≥6 weeks (or stopped for ≥2 weeks)	Allowed	1 generalized tonic-clonic seizure. Transient dizziness in 3 patients.
		Huang et al. (51)	44.94 ± 11.64 (active); 45.22 ± 10.85 (sham)	50.0%	Subjects and raters blind. Administrators blinding NR.	Anxiety Level (HARS)	PSQI, HDRS-24	Allowed if dose stable for ≥3 months	No. Exclusion	No severe adverse events. Mild headache (*n* = 8) and neck pain (*n* = 10), no significant difference between groups.
		Anderson et al. (43)	48 ± 8 (active); 46 ± 12 (sham)	44.8%	Subjects blind(#), administrator and raters blinding NR	Depression intensity (MADRS and HAD depression)	Other rating scale scores (HAD anxiety, CGI-S, CGI-I, GAF) and responder status at treatment end-point, scores at 12 weeks and treatment withdrawal.	Allowed	NR	1 hypomanic at 4 weeks + hospitalized for series of epileptic seizures 4 days after last treatment (active); 2 scalp discomfort
		Triggs et al. (59)	48.5 ± 10.8 (active right); 46.7 ± 15.3 (active left); 46.6 ± 20.2 (sham right); 41.9 ± 14.1 (sham left)	39.6%	Subjects and raters blind, administrators unblind	Depression intensity (HARS, BDI)	STAI	Yes, all patients continued their antidepressant medication throughout the study period.	NR	20 headaches (14 active), 13 scalp discomfort (10 active), 3 eye pain (3 active), 2 neck muscle soreness (2 active), 1 face spasm (active), 7 dizziness (5 active), 6 nausea (6 active), 8 fatigue (8 active), 4 insomnia (3 active), 2 impaired concentration (2 active),
		Deppermann et al. (46)	37.6 [19–63] (Active); 36.3 [22–56] (Sham); 33.4 [19–54] (Controls) (Mean [range])	38.8%	Subjects(*#) and raters blind. Administrators blinding NR.	PFC activation (fNIRS)	PAS, HARS and CAQ scales	Allowed if dose stable for ≥3 weeks	Yes, CBT (9 weeks)	NR
		Mantovani et al. (54)	40.2 ± 10.0 (Active); 39.8 ± 13.3 (Sham)	48.0%	Subjects(#), raters, and administrators blind	PD and depression intensity (PDSS and HDRS)	HARS-14, BDI-II, ZUNG-self administered scale, CGI-S, PGI, SASS	Allowed if dose stable for ≥4 weeks	Allowed if treatment as usual (unchanged ≥3 months)	No severe adverse event. Scalp pain, headache, neck pain. No cognitive subjective complaint. No significant difference between active and sham groups.
Primary outcome: anxiety symptoms intensity	TMS	Prasko et al. (56)	33.7 ± 9.2 (active); 33.8 ± 12.2 (sham)	26.7%	Subjects(*) and raters blind. Administrators not blind.	NR (HARS, PDSS, BAI and CGI rating scales	–	Yes, rTMS as add-on to SRIs for ≥6 weeks	NR	No severe adverse event, no headaches or cognitive difficulties
		Cohen et al. (44)	40.8 ± 9.9 (1 Hz); 41.8 ± 11.4 (10 Hz); 42.8 ± 14.8 (Sham)	70.8%	Subjects(*) and raters blind. Administrators not blind.	NR (PCL, TOP-8, HARS, HDRS and CAPS)	–	Allowed if dose stable for ≥3 weeks	Allowed if treatment as usual	No severe adverse event. Mild headaches (*n* = 14), neck pain (*n* = 2), diziness (*n* = 1). Manic episodes (*n* = 2; 1 in 1 Hz, 1 in 10 Hz), ear discomfort (*n* = 2), mild rage attack (*n* = 1)
		Leong et al. (52)	39.2 ± 13.5 (1 Hz); 43.5 ± 12.4 (10 Hz); 49.5 ± 6.9 (sham)	17.2%	Subjects and raters blind. Administrators not blind.	PTSD intensity (CAPS-IV)	HDRS-21, PCL, QIDS, BAI, GAD-7.	Allowed if dose stable for 4 weeks and during trial	Allowed if treatment as usual (no change allowed)	Suicidal ideations (*n* = 1); no other serious adverse event- minor events not reported.
		Watts et al. (60)	54.0 ± 12.3 (active); 57.8 ± 11.8 (sham)	90.0%	Subjects and raters blind. Administrators blinding NR.	NR (CAPS, PCL, BDI, STAI, BNCE measured)	–	Allowed if dose stable stable for ≥2 months	Allowed if treatment as usual (unchanged ≥2 months before rTMS onset)	NR
		Hermann et al. (49)	43.2 ± 12.6 (active); 46.6 ± 13.7 (sham)	43.2 ± 12.6 (active); 46.6 ± 13.7 (sham)	Subjects (#) and raters blind. Administrators blinding NR	Acrophobia intensity (AQ anxiety, AQ avoidance, BAT)	ATHQ, STAI, ASI3, PANAS, ADS	Not allowed	2 virtual reality exposure sessions (no involvement before trial allowed)	7 headaches (active), 1 drowsiness (active), 2 neckpain (sham), 1 pain at the stimulation area (sham)
	tDCS	de Lima et al. (53)	32.07 ± 6.5 (active); 29 ± 5.05 (sham)	36.7%	Subjects, raters, and administrators blind	Anxiety level (HARS + BAI)	ISSL, BDI, PANAS	Allowed if stable for ≥2 months	No. Exclusion	Active headache (30%), tingling (60%), sleepiness (90%); Sham: headache (35%), tingling (55%), sleepiness (20%)
		Movahed et al. (58)	28.7 ± 9.6	NR	Subjects blind(#), administrators not blind. Raters blinding NR	NR (PSWQ, HDRS-17, and HARS)	–	No. Exclusion	No. Exclusion	NR
		Nasiri et al. (55)	20.23 ± 2.89 (UP + tDCS); 21.53 ± 3.56 (UP); 20.53 ± 2.53 (wainting list)	25.6%	Subjects(*), raters, and administrators(*) blind	NR (ASI, BAI, BDI, GAD-Q-IV, IUS, PSWQ)	–	No. Exclusion	Yes. 12 sessions of UP	NR
		Dastjerdi et al. (45)	30.1 ± 7.09(tDCS- CES +); 34.1 ± 7.17 (tDCS + CES–); 30.2 ± 10.83 (tDCS + CES +)	43.3%	subjects blind, administrator and raters blinding NR	NR (BAI and BDI)	–	NR	NR	No serious adverse event
		Ahmadizadeh et al. (42)	44.50 ± 2.34 (Active); 43.00 ± 2.42 (Sham)	35.0%	Subjects (#), raters, and administrators (*) blind	PTSD intensity (PCL-5)	BAI, BDI	Allowed if dose stable for ≥2 months	Allowed if treatment as usual (unchanged ≥2 months)	No serious adverse event. No difference between active and sham group in mild effects. No individual data.
	DBS	Holtzheimer et al. (50)	50.53 ± 9.73 (active); 48.70 ± 0.56 (sham)	47.8%	Subject (#) and raters blind. Surgeons blind because surgery was before randomization Programmators unblind.	Rate of clinical response	MADRS, SAFTEE, DBS programming form, IDS-C30, QIDS-SR, WSAS, GAF, CGI, PGI, HAM-A, C-SSRS, HRSD-17, YMRS, QOL, and HLQ	Allowed if dose stable for ≥4 weeks and during trial	Allowed if treatment as usual (no change allowed)	Events at 6 months: 7 infections (6 active), 1 hospitalization (active), 1 suicide attempt (active), 1 suicidal ideation (active), 2 seizures (1 active), 1 hearing or visual disturbance (active), 1 local erosion over the pulse generator (active), 1 postoperative serious discomfort, 20 headaches (12 active), 41 pain/discomfort (26 active). Other side effects: persistent pain, anxiety, increasing in depressive symptoms, nausea/vomiting, sleep disturbance, paresthesia, dizziness, neuralgia.
		Puigdemont et al. (57)	42.0 ± 9.9 (OFF-ON *n* = 2); 50.7 ± 14.3 (ON-OFF *n* = 3)	NR	Subjects and raters blind.	Depression intensity (HAMD-17)	Remission/Relapse rates	Allowed and maintained stable during trial	NR	Not comprehensively described: include headache, dizziness, gastrointestinal disturbances, paresthesias, and advents associated with surgery and device.
Secondary outcome: disorder-specific scales	TMS	Ahmadizadeh et al. (61)	bilateral (52.10 ± 7.62), unilateral (51.89 ± 7.93), sham (47.5 ± 5.61)	100.0%	Subjects and raters blind. Administrators not blind	NS (PCL-M)	–	Allowed if dose stable ≥2 months	Allowed if treatment as usual (unchanged ≥2 months)	2 light headaches, 1 discomfort, 1 warmth sensation (number of events in active groups, but no significant difference between 3 conditions)
		Isserles et al. (62)	49 ± 12.5 (A), 40.5 ± 9.8 (B), 40.4 ± 10.5 (C) M ± SD	76.9%	Subjects and raters blind. Administrators blinding NR	PTSD intensity (CAPS)	PSS-SR, HDRS-24, BDI	Allowed if dose stable for ≥4 weeks	NR	1 tonic-clonic generalized seizure, mild headache, increased anxiety
		Kozel et al. (63)	(RTMS + withdrew = 31.18 ± 7.49) (Sham + Withdrew = 31.53 ± 6.33) (RTMS + completed = 34.06 ± 7.56) (Sham + completed = 32.93 ± 6.04)	NR	Subjects and raters blind. Administrators not blind	PTSD intensity (CAPS)	PCL	Allowed if dose stable during trial	Allowed if treatment as usual	No serious adverse event. Mild headaches (three participants, including 1 sham)
		Osuch et al. (64)	41.4 ± 12.3	11.1%	Subjects (*) and raters blind. Administrators not blind.	NS (CAPS, IES, HDRS, concurrent biological measures: 24 h urine collection and blood sample for hormonal dosing)	–	NR	rTMS as add-on to exposure therapy	NR
		Philip et al. (68)	active (48 ± 13); sham (53 ± 12)	84.0%	Subjects(#), administrators and raters blind.	PTSD intensity (CAPS)	PCL-5, IDS-SR, Q-LES-Q, SOFAS	Allowed if dose stable for ≥6 weeks	Allowed if treatment as usual (unchanged ≥6 weeks)	1 homicidal ideation (sham group); 1 suicidality (sham group); treatment site discomfort and headaches
		Philip et al. (65)	54.2 ± 12.0	73.3%	Subjects blind (#), administrators and raters blinding NR.	Depression and PTSD severity (QIDS and PCL)	Relationship between Intrinsic Alpha Frequency (IAF) à baseline and treatment outcomes, Adverse events	Allowed if dose stable for ≥6 weeks	Allowed if treatment as usual (unchanged ≥6 weeks)	No serious adverse event, *n* = 2 headaches (active); *n* = 1 nausea (active)
	tDCS	Smits et al. (66)	active (40.5 ± 10.6); sham (44.4 ± 9.4)	92.7%	Subjects and administrators blind. No raters blinding reported but autoevaluation on a weblink.	Inhibitory control training on the stop-signal task	Implicit association task, go/no-go task, stop signal response time, PCL-5, PANAS, STAXI-2, STAI-6, BDI-II, SSRT	Allowed if dose stable for ≥2 weeks	Allowed, randomization stratified on psychotherapy	No serious adverse event. Mild itching and burning sensations on the scalp (*p*'s < 0.001), light skin redness absent in the sham group (*p*=0.010). No significant difference in emotional state fluctuations.
		van't Wout-Frank et al. (67)	40.5 ± 8.8	100.0%	Subjects blind, administrators and raters blinding NR	Changes in skin conductance-based arousal and PCL-5	–	Allowed if dose stable for ≥6 weeks	Allowed if treatment as usual (unchanged ≥6 weeks)	NS

*AQ, Acrophobia Questionnaire; ASI, anxiety sensitivity index; BAI, Beck anxiety inventory; BDI, Beck depression inventory; BNCE, Brief Neurobehavioral Cognitive Examination; C-SSRS, Columbia Suicide Severity Rating Scale; CAPS, clinician-administered PTSD scale; CAQ, cardiac anxiety questionnaire; CGI, clinical global impression scale (s-severity; i-improvement); DASS-DEP, depression anxiety stress scale; fNIRS, functional near-infrared imaging spectrophotometry; GAD-7, generalized anxiety disorder- 7 items; GAD-Q-IV, generalized anxiety disorder questionnaire IV; GAF, global assessment of functioning; HAD, Hospital anxiety and depression scale; HARS, Hamilton anxiety rating scale; HDRS, Hamilton depression rating scale; HLQ, Health and Labor Questionnaire; IDS-C30, inventory of depressive symptomatology; ISSL, Lipp inventory of stress symptoms for adults; ITT, intent to treat; IUS, intolerance of incertainty scale; MADRS, Montgomery Asberg depression rating scale; NR, not reported; ns, not significant; PANAS, positive and negative affect schedule; PAS, panic and agoraphobia scale; PCL, PTSD checklist; PDSS, panic disorder severity scale; PFC, prefrontal cortex; PGI, patient global impression; PP, per protocole; PSQI, Pittsburgh sleep quality index; PSWQ, Penn state worry questionnaire; QIDS, Quick inventory of depressive symptomatologie; QOL, Quality of Life Enjoyment and Satisfaction Questionnaire; SAFTEE, Systematic Assessment for Treatment Emergent Events; SASS, self- reported social adaptation scale; SRI, serotonin reuptake inhibitor; STAI-S, STAI state; STAI-T, STAI trait; STAI, state trait anxiety inventory; TOP-8, treatment outcome PTSD Scale; WSAS, work and social adjustment scale; YMRS, Young mania rating scale.*

* *Condition easily distinguishable (Supplementary Table 1).*

# *Subjects blinding assessment (Supplementary Table 1)*.

**Table 4:** Raw data of psychometrics in included studies.

**Outcome in meta-analysis**	**Technique**	**References**	**Study design**	**Scale(s) included in analysis**	**Score pre-treatment**			**Score posttreatment**			**Score at end of the study follow-up**			**Follow-up duration (post-treatment)**
					**Group A**	**Group B**	**Group C**	**Group A**	**Group B**	**Group C**	**Group A**	**Group B**	**Group C**	
	TMS	Diefenbach et al. (47) (GAD)	Single center RCT, 1 Hz Right dlPFC rTMS (group A, *n* = 13) vs. Sham (group C, *n* = 12)	HARS	25.31 ± 5.23	–	20.75 ± 3.72	12.10 ± 5.77	–	14.38 ± 4.78	10.36 ± 7.86	–	17.95 ± 7.48	3 months [*n* = 12, *n*(C) = 13]
				PSWQ	69.54 ± 5.77	–	62.08 ± 9.58	61.73 ± 8.8	–	61.77 ± 8.35	54.36 ± 8.10	–	57.49 ± 8.85	
		Dilkov et al. (48) (GAD)	Two-center RCT, 20 Hz Right dlPFC rTMS (group A, *n* = 15) vs. Sham (group C, *n* = 25)	HARS	15 ± 1	–	14 ± 3	4 ± 1	–	14 ± 6	4 ± 1	–	15 ± 4	4 weeks [*n*(A) = 15, *n*(C) = 25]
		Huang et al. (51) (GAD)	Single center RCT, 1 Hz Right PPC rTMS (group A, *n* = 18) vs. Sham (group C, *n* = 18)	HARS	20.78 ± 4.04	–	20.33 ± 2.77	11.67 ± 5.93	–	18.72 ± 4.56	10.89 ± 5.99	–	17.28 ± 5.07	4 weeks [*n*(A) = 18, *n*(C) = 18]
				PSQI	12.61 ± 2.85	–	13.06 ± 4.26	7.06 ± 2.75	–	11.44 ± 4.13	7.28 ± 3.37	–	11.56 ± 3.82	
		Anderson et al. (43) (MDD)	Single center RCT, 10 Hz Left dlPFC rTMS (group A, *n* = 13) vs. Sham (group C, *n* = 16)	HAD (anxiety)	13.8 ± 4.0	–	14.1 ± 5.1	9.9 ± 4.9	–	11.9 ± 5.2	9.2 ± 4.9	–	10.1 ± 4.9	2 months [*n*(A) = 9, *n*(C) = 13]
				HAD (depression)	14.6 ± 3.3	–	15.1 ± 3.0	9.7 ± 5.5	–	14.2 ± 4.2	8.3 ± 5.6	–	13.6 ± 3.7	
				MADRS	26.7 ± 3.6	–	27.7 ± 7.1	15 ± 9.7	–	23.4 ± 9.8	14.0 ± 11.5	–	21.9 ± 9.7	
		Triggs et al. (59) (MDD)	Singler center RCT, 5 Hz Left dlPFC rTMS (group A, *n* = 18) vs. 5 Hz right dlPFC rTMS (group B, *n* = 16) vs. Sham right or left (group C, *n* = 14)	STAI-S	5.2 ± 13.0	52.3 ± 12.6	53.5 ± 9.5	45.8 ± 12.9	39.4 ± 13.0	46.3 ± 13.3	45.8 ± 14.9	40.0 ± 14.8	47.4 ± 15.4	3 months [*n* = 45]
				STAI-T	60.1 ± 11.3	60.3 ± 9.4	58.6 ± 7.3	56.1 ± 13.1	48.5 ± 15.1	55.2 ± 11,9	51.3 ± 15.8	47.7 ± 16.3	52.9 ± 14.8	
				HDRS	28.2 ± 6.0	27.2 ± 4.8	27.5 ± 3.0	19.8 ± 9.1	13.7 ± 7.6	17.7 ± 10.4	16.3 ± 11.5	11.7 ± 9.3	17.9 ± 11.6	
				BDI	29.8 ± 10.9	32.3 ± 8.7	28.8 ± 6.7	18.7 ± 12.0	18.7 ± 12.0	18.7 ± 12.0	17.8 ± 14.6	17.7 ± 15.0	19.4 ± 14.0	
		Deppermann et al. (46) (PD)	Two-center RCT, Left dlPFC iTBS (group A, *n* = 22) vs. Sham (group C, *n* = 22) as add-on for psychoeducation. Additional healthy controls (group B, *n* = 23)	HARS	22.41 ± 8.97	3.83 ± 3.20	23.0 ± 7.10	18.37 ± 10.05	2.74 ± 3.57	15.20 ± 8.81	–	–	–	–
				PAS	20.76 ± 7.76	0.22 ± 1.04	20.52 ± 8.10	14.91 ± 6.90	0.13 ± 0.34	15.34 ± 8.30	–	–	–	
		Mantovani et al. (54) (PD + MDD)	Single center RCT, 1 Hz Right dlPFC rTMS (group A, *n* = 12) vs. Sham (group C, *n* = 13)	HARS	29.1 ± 8.7	–	25.3 ± 10.6	21.0 ± 9.2	–	20.8 ± 7.1	NR	–	NR	6 months (only for responders) [*n* = 8]
				PDSS	18.9 ± 2.9	–	17.1 ± 3.9	10.4 ± 6.5	–	16.7 ± 4.2	NR	–	NR	
				HDRS	31.9 ± 6.5	–	31.1 ± 8.3	25.3 ± 9.8	–	26.8 ± 9.8	NR	–	NR	
		Prasko et al. (56) (PD)	Single center RCT, 1 Hz Right dlPFC rTMS (group A, *n* = 7) vs. Sham (group C, *n* = 8) as add–on for SSRI	HARS	21.43 ± 4.791	–	21.13 ± 5.111	18.43 ± 11.41	–	13.3 ± 6.175	15.86 ± 4.914	–	10.75 ± 3.845	2 weeks [*n* = NR]
				BAI	34.86 ± 10.07	–	25.38 ± 14.21	24.14 ± 11.57	–	15.63 ± 7.891	23.86 ± 10.43	–	14.5 ± 6.164	
				PDSS	17.86 ± 3.338	–	16.25 ± 4.464	14.57 ± 4.429	–	10.75 ± 6.431	11.71 ± 4.071	–	8.25 ± 4.95	
		Cohen et al. (44) (PTSD)	Single center RCT, 1 Hz (group A, *n* = 8) or 10 Hz Right dlPFC rTMS (group B, *n* = 10) vs. Sham (group C, *n* = 6)	HARS	19.3 ± 4.2	21.8 ± 5.6	19.2 ± 6.7	18.8 ± 4.9	11.8 ± 3.9	18.0 ± 4.8	19.0 ± 3.8	16.2 ± 5.8	16.2 ± 5.5	2 weeks [*n*(A) = 8, *n*(B) = 10, *n*(C) = 6]
				CAPS	87.9 ± 17.5	94.5 ± 15.8	84.3 ± 19.3	Not measured	Not measured	Not measured	87.9 ± 17.5	64.0 ± 16.1	74.8 ± 9.7	
				PCL	62.5 ± 7.8	63.2 ± 13.1	56.8 ± 3.6	56.0 ± 9.5	43.5 ± 8.3	55.0 ± 4.9	58.7 ± 9.3	46.3 ± 10.8	53.0 ± 5.5	
				TOP-8	22.9 ± 2.3	22.3 ± 6.6	18.3 ± 6.0	19.5 ± 4.2	12.9 ± 4.5	18.0 ± 3.2	21.0 ± 3.8	17.2 ± 6.3	15.6 ± 3.2	
		Leong et al. (52) (PTSD)	Single center RCT, 1 Hz (group A, *n* = 11) or 10 Hz Right dlPFC rTMS (group B, *n* = 9) vs. Sham (group C, *n* = 9)	BAI	34.60 ± 18.46	30.22 ± 20.63	35.14 ± 10.86	24.70 ± 18.19	22.77 ± 18.78	28.14 ± 10.97	26.55 ± 15.35	28.62 ± 19.33	33.20 ± 11.51	3 months [*n*(A) = 7, *n*(B) = 7, *n*(C) = 3]
				CAPS	72.27 ± 25.34	69.44 ± 18.29	55.22 ± 13.17	59.80 ± 35.83	74.00 ± 30.97	65.12 ± 14.97	55.40 ± 29.20	70.55 ± 27.98	71.50 ± 19.97	
				PCL	59.40 ± 16.44	65.33 ± 11.40	61.62 ± 7.96	48.10 ± 23.54	53.44 ± 22.80	52.14 ± 10.05	48.66 ± 17.25	57.12 ± 14.77	52.66 ± 13.44	
		Watts et al. (60) (PTSD)	Single center RCT, 1 Hz Right dlPFC rTMS (group A, *n* = 10) vs. Sham (group C, *n* = 10)	STAI	57.3 ± 10.9	–	54.5 ± 6.1	47.4 ± 13.4	–	52.2 ± 5.6	IC	–	IC	2 months [*n* = NR]
				CAPS	81.6 ± 9.5	–	72.3 ± 12.2	53.9 ± 15.3	–	61.7 ± 11.1	64.2	–	IC	
				PCL	64.9 ± 6.5	–	57.3 ± 3.7	48.7 ± 9.9	–	54.8 ± 5.0	IC	–	IC	
		Hermann et al. (49) (SP)	Single RCT, 10 Hz mPFC rTMS (group A, *n* = 20) vs. Sham (group C, *n* = 19), as add on to 2 virtual-reality exposure sessions.	STAI-X2	39.31 ± 7.32	–	35.63 ± 5.26	36.56 ± 7.16	–	33.58 ± 5.55	35.75 ± 7.33	–	35.58 ± 6.91	3 months [*n*(A) = 20, *n*(C) = 19]
				AQ-anxiety	53.7 ± 18.3	–	50.5 ± 18.1	36.3 ± 18.7	–	43.2 ± 19.4	NR	NR	NR	
				AQ-avoidance	33.5 ± 6.4	–	32.6 ± 5.8	27.7 ± 5.2	–	30.0 ± 6.2	NR	NR	NR	
	tDCS	de Lima et al. (53) (GAD)	Two-center RCT, tDCS 2 mA (cathode Right supraorbital cortex, anode Left dlPFC) (group A, *n* = 15) vs. Sham (group C, *n* = 15)	HARS	31.47 ± 14.202	–	26.93 ± 13.199	19.60 ± 11.758	–	20.13 ± 14.232	16.27 ± 11.603	–	17.87 ± 14.172	1 week [*n*(A) = 15, n(C) = 15]
				BAI	32.27 ± 17.854	–	24.67 ± 11.635	18.40 ± 15.665	–	17.47 ± 13.679	18.20 ± 15.708	–	16.60 ± 13.579	
				ISSL	10.27 ± 5.775	–	9.27 ± 4.906	5.47 ± 4.518	–	6.20 ± 4.854	6.67 ± 4.203	–	7.33 ± 4.835	
	tDCS	Movahed et al. (58) (GAD)	Single center RCT, 2 mA tDCS (group A, *n* = 6) vs. pharmacotherapy (group B, *n* = 6) vs. Sham (group C, *n* = 6)	HARS	44.32 ± 5.50	44 ± 8.69	42.83 ± 13.10	36 ± 3.57	35.33 ± 7.06	43.33 ± 12.89	37.0 ± 4.09	36.50 ± 5.43	44.16 ± 13.31	2 months [*n* = NR]
				PSWQ	47 ± 6	46 ± 8.57	46 ± 9.01	38.33 ± 6.05	29.16 ± 6.30	46.66 ± 8.31	38.66 ± 4.80	31.66 ± 4.08	46.50 ± 8.87	
		Nasiri et al. (55) (GAD + MDD)	Single center RCT, tDCS + UP (group A, *n* = 13) vs. UP alone (group B, *n* = 15) vs. waiting list as control (group C, *n* = 15)	BAI	34.15 ± 7.53	34.27 ± 10.02	33.60 ± 7.76	9.54 ± 5.82	17.60 ± 8.93	34.00 ± 6.50	8.54 ± 4.75	14.80 ± 7.37	36.73 ± 5.82	3 months [*n*(A) = 13, *n*(B) = 15, *n*(C) = 15]
				BDI	28.61 ± 6.99	27.60 ± 8.68	28.13 ± 11.06	8.08 ± 5.22	9.80 ± 7.08	27.60 ± 9.16	6.85 ± 4.10	9.80 ± 5.03	28.47 ± 5.91	
				GAD-Q-IV	25.77 ± 3.44	26.07 ± 4.13	24.80 ± 2.30	10.54 ± 2.44	11.87 ± 3.52	25.87 ± 2.42	8.61 ± 2.36	10.53 ± 3.25	27.27 ± 2.96	
				PSWQ	65.61 ± 7.12	63.33 ± 7.53	60.40 ± 11.49	35.08 ± 5.33	41.33 ± 4.99	20.27 ± 7.76	31.61 ± 4.41	39.73 ± 6.13	65.07 ± 10.09	
		Dastjerdi et al. (45) (MDD)	Single center RCT, Active tDCS + Active CES (group 1, *n* = 10) vs. Active tDCS + Sham CES (group B, *n* = 10) vs. Sham tDCS + Active CES (group C, *n* = 10)	BAI	15.00 ± 5.84	14.1 ± 6.6	18.4 ± 5.2	9.90 ± 5.3	2.2 ± 5.1	7.0 ± 3.1	–	–	–	–
				BDI	28.2 ± 6.1	25.7 ± 6.7	26.2 ± 7.6	16.70 ± 4.93	10.00 ± 6.6	8.90 ± 1.5	–	–	–	–
		Ahmadizadeh et al. (42) (PTSD)	Single center RCT, tDCS 2 mA cathode Right dlPFC, anode Left dlPFC (Group A, *n* = 18) vs. Sham (Group C, *n* = 16)	BAI	26.61 ± 7.58	–	32.12 ± 4.42	20.94 ± 6.38	–	31.43 ± 4.90	21.44 ± 8.24	–	33.62 ± 5.03	1 month [*n*(A) = 18n(C) = 16]
				PCL	54.39 ± 2.93	–	54.81 ± 2.79	37.44 ± 10.77	–	52.00 ± 11.19	41.28 ± 8.86	–	52.81 ± 5.07	
	DBS	Holtzheimer et al. (50) (MDD)	Multicenter (20) RCT, bilateral subcallosal cingulate active DBS (group A, *n* = 56) vs. Sham (group C, n = 29)	HARS	(*n* = 40) 15.9 ± 6.52	–	(*n* = 19) 16.7 ± 6.67	(*n* = 40) 15.0 ± 7.54	–	(*n* = 19) 18.6 ± 8.70	(*n* = 40) 12.3 ± 7.99	–	(*n* = 19) 14.2 ± 7.97	6 months (until 30 months but anxiety NR)
				MADRS	33.8 ± 4.5	–	37.3 ± 3.8	27.2 ± 11.3	–	30.0 ± 10.6	23.2 ± 12.2	–	26.7 ± 12.1	
				HDRS	20.3 ± 3.8	–	22.6 ± 4.4	17.5 ± 7.6	–	19 ± 7.9	–	–	–	
		Puigdemont et al. (57) (MDD)	Single center RCT, bilateral subcallosal cingulate gyrus DBS vs. Sham, cross-over design, *n* = 5, after DBS-obtained remission	HDRS (anxiety item)	1.4 ± 0.548	–	–	2.6 ± 3.715	–	–	–	–	–	–
Secondary outcome: disorder-specific scales	TMS	Ahmadizadeh et al. (61) (PTSD)	Single center RCT, 20 Hz Right dlPFC rTMS (group A, *n* = 19) vs. 20 Hz Bilateral dlPFC rTMS (group B, *n* = 19) vs. Sham (group C, *n* = 20)	PCL-M	70.57 ± 9.00	71.26 ± 7.65	70.55 ± 9.04	49.41 ± 6.53	45.81 ± 4.67	66.93 ± 10.34	–	–	–	–
		Isserles et al. (62) (PTSD)	Single center RCT, Active 20 Hz mPFC dTMS + Active Exposure (group A, *n* = 9) vs. Active dTMS + Sham exposure (group B, *n* = 8) vs. Sham dTMS + active exposure (group C, *n* = 9)	CAPS	88 ± 5.5	86 ± 5.4	86 ± 9.2	61 ± 8.8	76 ± 10.9	76 ± 10.7	–	–	–	–
		Kozel et al. (63) (PTSD)	Single center RCT, 1 Hz Right dlPFC rTMS (group A, *n* = 54) vs. Sham (group C, *n* = 49) as add-on for CPT	CAPS	75.28 ± 3.24	–	73.88 ± 3.51	45.95 ± 4.04	–	53.86 ± 4.66	27.51 ± 4.04	–	37.57 ± 4.49	6 months [*n*(A)=31 *n*(C)=28]
				PCL	55.93 ± 1.83	–	52.82 ± 2.05	39.25 ± 2.20	–	42.35 ± 2.53	30.87 ± 2.15	–	38.08 ± 2.46	
		Osuch et al. (64) (PTSD)	Single center RCT, 1 Hz Right dlPFC rTMS vs. Sham, cross-over design, as add on to exposure therapy	CAPS	4.55 ± 1.69	–	4.4 ± 0.7	4.9 ± 1.37	–	5.0 ± 1.25	NR	–	–	–
		Philip et al. (68) (PTSD)	Single center RCT, right dlPFC iTBS (group A, *n* = 25) vs. Sham (group C, *n* = 25)	CAPS	47.9 ± 10.0	–	47.4 ± 10.6	38.6 ± 11.4	–	39.4 ± 13.8	NR	–	NR	1 month [*n*(A)=24n(C)=20]
				PCL	49.4 ± 9.4	–	50.0 ± 11.4	35.5 ± 13.9	–	39.4 ± 16.8	NR	–	NR	
		Philip et al. (65) (PTSD)	Two centers RCT, synchronized TMS on IAF (group A, *n* = 10) vs. Sham (group C, *n* = 13) (+ 4 weeks of optional unblinded sTMS)	PCL	50.1 ± 7.4	–	49.2 ± 9.3	38.1 ± 17.0	–	39.1 ± 14.9	30.5 ± 12.8	–	37.1 ± 17.4	1 month [*n* = NR]
				QIDS	16.1 ± 3.2	–	15.9 ± 2.7	10.7 ± 5.7	–	11.3 ± 3.8	11.2 ± 5.0	–	11.5 ± 4.7	
	tDCS	Smits et al. (66) (PTSD)	Single center RCT, right IFG 1.25 mA tDCS (group A, *n* = 23) vs. Sham (group C, *n* = 23)	PCL	2.45 ± 0.55	–	2.19 ± 0.63	2.02 ± 0.72	–	2.07 ± 0.72	1.62 ± 0.48	–	1.46 ± 0.92	1 year [*n*(A)=24 *n*(C)=33]
		Van't Wout-Frank et al. (67)	Single RCT, 2 mA on vmPFC tDCS (group A, *n* = 6/12) vs. Sham (group C, *n* = 6/12)	PCL	41.83 ± 10.6	–	44.33 ± 15.5	32.5 ± 16.3	–	35.8 ± 16.2	29.0 ± 13.4	–	35.3 ± 19.6	1 month [*n* = NR]

***Intervention:** CES, cranial electrical stimulation; DBS, deep brain stimulation; dTMS, deep TMS; iTBS, intermittent theta burst stimulation; SSRI, serotonin selective reuptake inhibitor; sTMS, synchronized TMS; tDCS, transcranial direct current stimulation; TMS, transcranial magnetic stimulation; UP, Unified Protocol for Transdiagnostic Treatment of Emotional Disorders.*

***Disorders:** GAD, generalized anxiety disorder; PD, panic disorder; MDD, major depressive disorder; PTSD, post-traumatic stress disorder; SP, specific phobia; TR, treatment-resistant; TRD, treatment-resistant depression.*

***Targets:** PFC, prefrontal cortex; dlPFC, dorso-lateral PFC; vmPFC, ventromedial PFC; mPFC, medial PFC; PPC, posterior parietal cortex; IFG, inferior frontal gyrus; -IC, incomplete data; NR, not reported; ns, not significant; RCT, randomized controlled trial.*

***Psychometric scales:** AQ, Acrophobia Questionnaire; BAI, Beck anxiety inventory; BDI, Beck depression inventory; CAPS, clinician-administered PTSD scale; GAD-Q-IV, generalized anxiety disorder questionnaire IV; HAD, Hospital anxiety and depression scale; HARS, Hamilton anxiety rating scale; HDRS, Hamilton depression rating scale; ISSL, Lipp inventory of stress symptoms for adults; MADRS, Montgomery Asberg depression rating scale; PAS, panic and agoraphobia scale; PCL, PTSD checklist; PDSS, panic disorder severity scale; PSQI, Pittsburgh sleep quality index; PSWQ, Penn state worry questionnaire; QIDS, Quick inventory of depressive symptomatologie; STAI, state trait anxiety inventory; STAI-S, STAI state; STAI-T, STAI trait; TOP-8, treatment outcome PTSD Scale*.

### Secondary Outcomes

#### Rates of Clinically Significant Response and Remission

**Figure 5:**
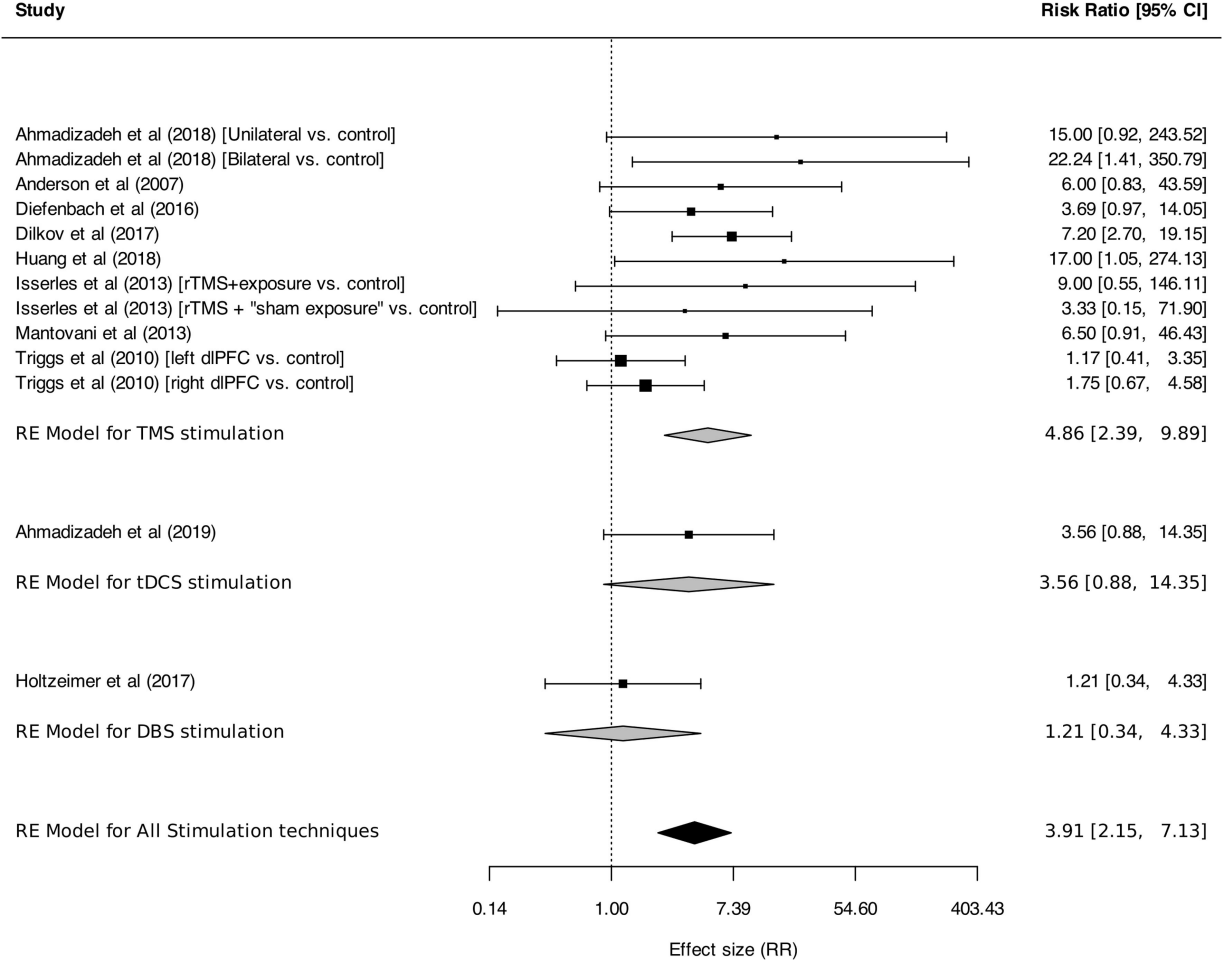
Response rates for anxiety, stressor-related disorders and MDD, subgroups by stimulation technique. Statistics: TMS: heterogeneity Sigma1^2^ = 0.42, Sigma2^2^ = 0.00, Q(df = 10) = 13.36, *p* = 0.20; *I*^2^ = 39%; test for overall effect: *Z* = 4.35 (*p* < 0.0001); tDCS: heterogeneity: not applicable. DBS: heterogeneity: not applicable. Total: heterogeneity Sigma1^2^ = 0.36, Sigma2^2^ = 0.00, Q(df = 12) = 15.84, *p* = 0.20; *I*^2^ = 37%; test for overall effect: *Z* = 4.45 (*p* < 0.0001); F(df1 = 2, df2 = 22) = 0.05, *p* = 0.95.

**Figure 6:**
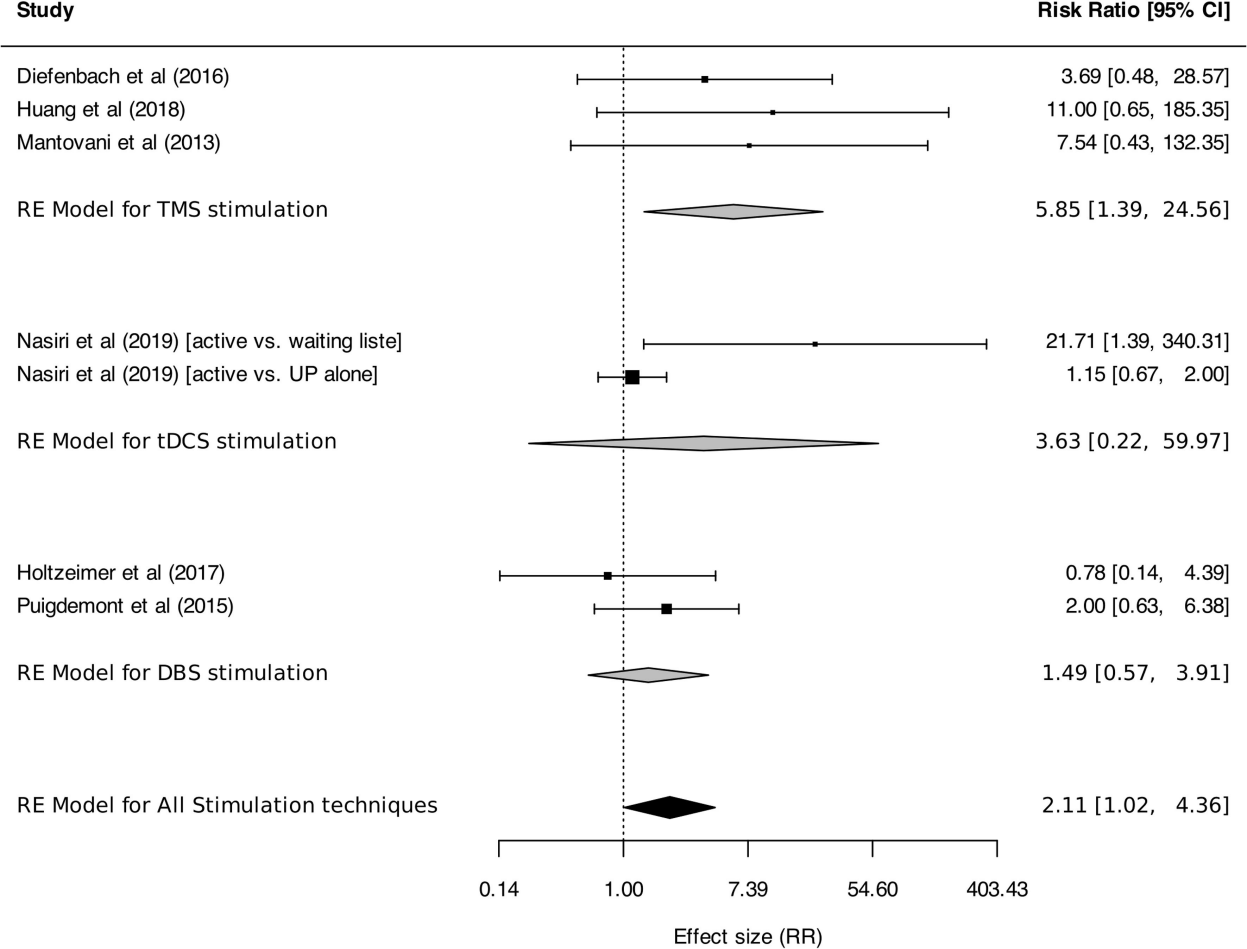
Remission rates for anxiety, stressor-related disorders and MDD, subgroups by stimulation technique. Statistics: TMS: heterogeneity Sigma1^2^ = 0.00, Sigma2^2^ = 0.00, Q(df = 2) = 0.42, *p* = 0.81; *I*^2^ = 0%; test for overall effect: *Z* = 2.41 (*p* = 0.02); tDCS: heterogeneity Sigma1^2^ = 0.00, Sigma2^2^ = 3.28, Q(df = 1) = 4.20, *p*-val = 0.04; *I*^2^ = 76%; test for overall effect: *Z* = 2.41 (*p* = 0.02); DBS: heterogeneity Chi^2^ = 0.79, df = 1 (*p* = 0.37); *I*^2^ = 0%; test for overall effect: *Z* = 0.90 (*p* = 0.37); Total: heterogeneity Sigma1^2^ = 0.00, Sigma2^2^ = 0.29, Q(df = 6) = 9.15, *p* = 0.17; *I*^2^ = 33%; test for overall effect: *Z* = 2.01 (*p* = 0.04); test for subgroup differences: F(df1 = 2, df2 = 4) = 0.97, *p* = 0.45.

Meta-analysis was performed on response and remission rates, as defined by the authors. Criteria were alternatively defined on the basis of generic or disorder-specific scales (see Table 2). Overall, neurostimulation was associated with higher response rates than control interventions [RR: 3.91 (95% CI, 2.15–7.13), *I*^2^ = 31%]. This result was found in TMS studies [RR: 4.86 (95% CI, 2.39–9.89), *I*^2^ = 39%] but was not statistically significant for tDCS [RR: 3.56 (95% CI, 0.88–14.35)] or DBS [RR: 1.21 (95% CI, 0.34–4.33); Figure 5]. Anxiety remission rates were higher after neurostimulation overall [RR: 2.11 (95%CI, 1.02–4.36), *I*^2^ = 33%], and also after TMS [RR: 5.85 (95% CI, 1.39–24.56), *I*^2^ = 0%], but not after tDCS [RR: 3.63 (95% CI, 0.22–59.97), *I*^2^ = 76%], and DBS [RR: 1.49 (95% CI, 0.57–3.91), *I*^2^ = 0%; Figure 6].

#### Follow-Up Results

Data of follow-up visits were not submitted to meta-analysis because of a high heterogeneity in the length of follow-up periods, data reporting and attrition rates (see Table 4 for detailed reporting).

For TMS, three studies stopped follow-up visits after 2–4 weeks (44, 51, 56), 7 after 1–3 months (43, 47–49, 52, 59, 60), and one at 6 months (54). Four studies showed persistent differences between active and sham groups at the end of follow-up in favor of neurostimulation (47, 48, 51, 54) and two reported data suggesting the persistence of differences between groups, although statistics were not presented (52, 59). There was no statistically significant difference between groups in two trials (43, 49). Finally, one article reported incomplete data (60).

In tDCS studies, follow-up visits were also conducted at different time points: 1 week (53), 4 weeks (42), 2 months (58), and 3 months (55). Three studies described a persistently significant difference between active and sham groups (42, 55, 58). One trial presented within-group comparisons, but no statistics were provided for between-group comparisons at the end of follow-up (53).

In DBS studies, all subjects underwent an unblinded treatment phase following the endpoint evaluation. Thereafter, they received an active stimulation for six consecutive months (50), or until loss to follow-up (57). Values of HARS were similar between treatment groups at 6 months follow-up in both studies.

#### Efficacy by Disorder as Measured With Disorder-Specific Scales

**Figure 7:**
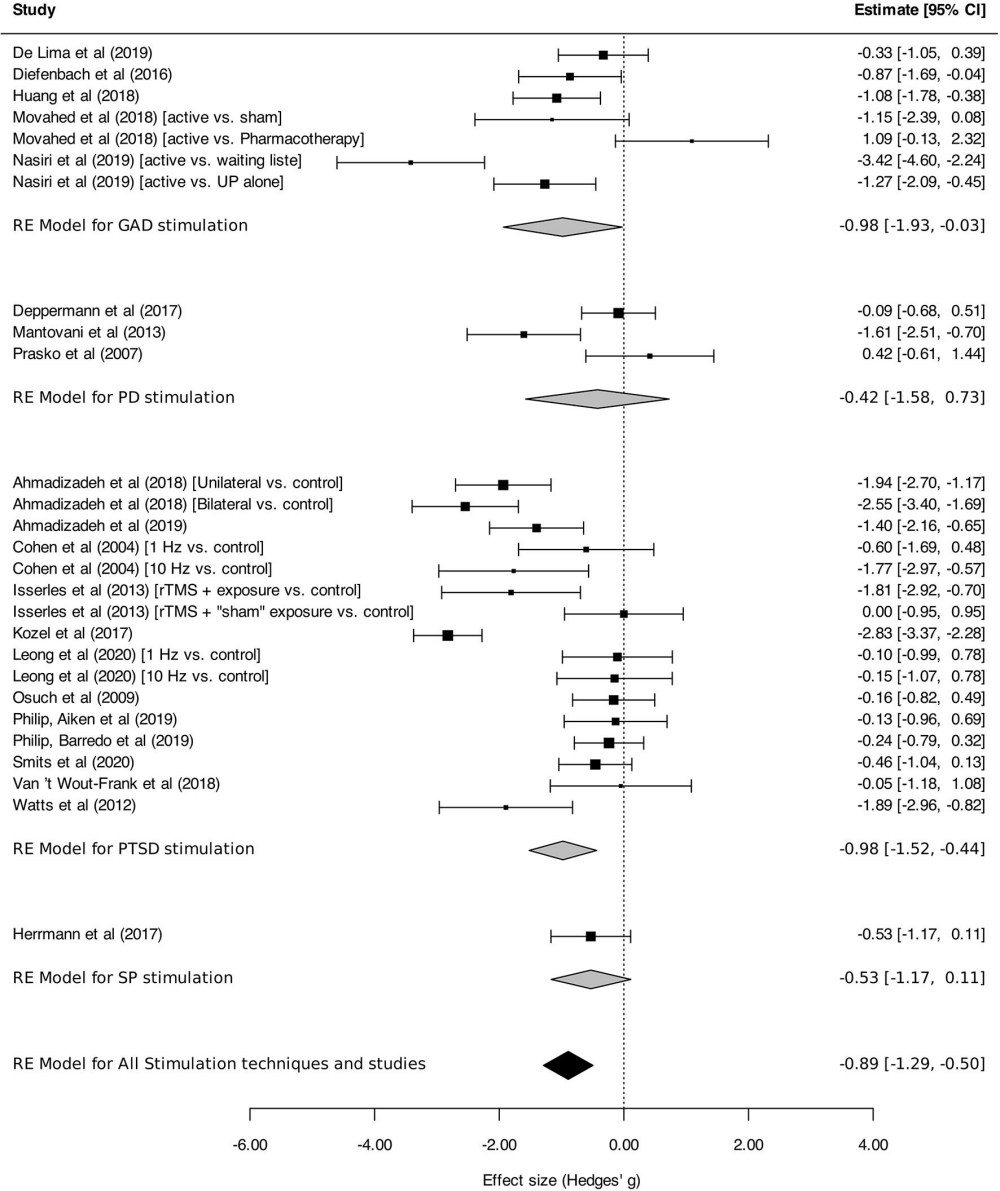
Change in the severity of anxiety, stressor-related disorders, assessed by disorder-specific scale. Statistics: GAD: heterogeneity Sigma1^2^ = 0.19, Sigma2^2^ = 1.13; Q(df = 6) = 31.04, *p* < 0.0001; *I*^2^ = 86%; test for overall effect: *Z* = −2.02 (*p* = 0.04); PD: heterogeneity Sigma1^2^ = 0.42; Sigma2^2^ = 0.42; Q(df = 2) = 10.22, *p* = 0.006; *I*^2^ = 82%; test for overall effect: *Z* = −0.72 (*p* = 0.47); PTSD: heterogeneity Sigma1^2^ = 0.51; Sigma2^2^ = 0.32; Q(df = 15) = 103.78, *p* < 0.0001; *I*^2^ = 83%; test for overall effect: *Z* = −3.52 (*p* = 0.0004); SP: heterogeneity : not applicable; test for overall effect: *Z* = −1.63 (*p* = 0.10); Total: heterogeneity Sigma1^2^ = 0.25, Sigma2^2^ = *0.57*; Q(df = 26) = 153.87, *p* < 0.0001; *I*^2^ = *83*%; test for overall effect: *Z* = –*4.40* (*p* < 0.0001); test for subgroup differences: F(df1 = 3, df2 = 23) = 0.29, *p* = 0.83.

Figure 7 presents the efficacy of neurostimulation on reducing the severity of anxiety and stressor-related disorders, assessed by disorder-specific scales. Significant reduction of symptom severity was found for neurostimulation in the whole sample of trials [SMD, −0.89 (95%CI, −1.29 to −0.50), *I*^2^ = 83%]. However, in the subgroup analysis, a significant reduction of symptom intensity in favor of neurostimulation was only found for GAD [SMD, −0.98 (95% CI, −1.93 to −0.03), *I*^2^ = 86%] and PTSD [SMD, −0.98 (95% CI, −1.52 to −0.44), *I*^2^ = 83%].

#### Adverse Events

Data regarding adverse events were mostly reported irrespective of intervention groups (active or control). They are therefore reported in a descriptive manner (see Supplementary Material for a more detailed reporting). For TMS studies, serious adverse events comprised three cases of generalized tonic-clonic seizures in active groups (43, 48, 62), two suicidal ideation episodes in the active group (52), one homicidal ideation emergence, one hospitalization for suicidality during follow-up [both receiving sham stimulation; (68)], two manic and one hypomanic episodes [all receiving active stimulation; (43, 44)], and one chest pain occurrence in the active group, which was considered to be unrelated to the intervention (47). No serious adverse event was reported for tDCS. Other adverse events were mild, including: (i) TMS: headaches, pain at the stimulation point, neck, face (when specified, there was no significant difference between active and sham conditions for these side effects), dizziness/nausea, ipsilateral facial twitch, and ear discomfort; (ii) tDCS: headache, tingling, sleepiness, mild itching or burning sensations on the scalp and light skin redness. Only sleepiness, sensations on the scalp and skin redness were more frequently reported in active groups.

Side effects were reported in only one of the two DBS studies (50). Serious adverse events at 6 months were infections (six patients in the active stimulation group, one sham) related to the study device or surgery, hospitalization (one active) for unspecified reason, suicide attempt (one active), suicidal ideation (one active), seizures (one active, one sham), hearing or visual disturbances (one active), local erosion over the pulse generator (one active), serious postoperative discomfort (8). The most frequent adverse events were mild, including headache, acute pain/discomfort, persistent pain, anxiety, increase in depressive symptom intensity, nausea/vomiting, sleep disturbances, paresthesia, and dizziness (50).

### Meta-Regression Analysis

In the meta-regression analysis, the difference in pre/post-treatment changes of depression scores between active and sham arms (Coefficient = 0.86, *p* = 0.0003) significantly accounted for the heterogeneity [heterogeneity test QE(df = 19) = 19.6, *p* = 0.42]. No statistically significant link was found for other covariates, namely: the baseline anxiety scores, the changes in PTSD scores, the gender ratio and the mean age of participants, the total number of sessions during the protocol and, in the TMS studies, the total number of pulses and the percentage of motor threshold did not explain results heterogeneity. Statistical characteristics for each factor are reported in the Table 5.

**Table 5:** Meta-regression analysis.

**Moderator**	**Coefficient**	**SE**	**t**	***p***	**95%CI**
Gender ratio	−0.01	0.01	0.52	0.61	(−0.03 to 0.02)
Mean age	0.01	0.02	0.61	0.55	(−0.03 to 0.06)
Motor Treshold percentage	0.01	0.02	0.32	0.75	(−0.03 to −0.05)
Number of sessions	−0.06	0.03	−2.04	0.05	(−0.12 to 0.00)
Pulses	−0.00	0.00	−2.68	0.02	(−0.00 to −0.00)
SMD anxiety baseline (active vs. sham)	−0.40	0.51	−0.79	0.44	(−1.46 to 0.65)
SMD depression pre/post treatment (active vs. sham)	0.86	0.20	4.40	0.0003	(0.45 to 1.27)
SMD ptsd pre/post treatment (active vs. sham)	0.80	0.68	1.17	0.33	(−1.37 to 2.97)

### Risk of Bias and Quality Assessment

#### Publication Bias

**Figure 8:**
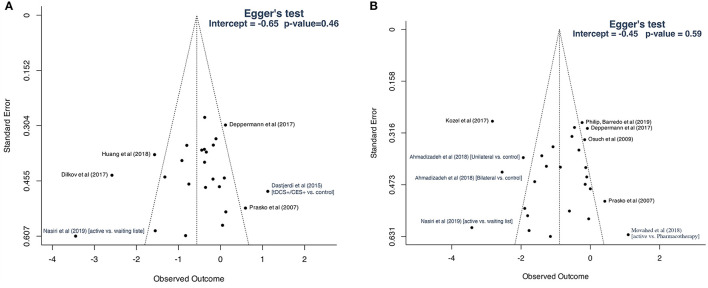
Funnel plots: **(A)** studies included for primary outcome; **(B)** studies included for secondary analysis based on disorder-specific scales.

Visual inspection of the funnel plot of studies included for primary outcome analysis revealed an asymmetric distribution of studies, indicating that publication bias may have occurred (Figure 8A). However, Egger's test was not indicative of a statistically significant bias (*p* = 0.46). There was no evidence of publication bias for studies included for the analysis of disorders-specific scales (Figure 8B; *p* = 0.59).

#### Risk of Bias in Methodology

Detailed evaluation of the risk of bias is reported in Supplementary Table 1. The majority of studies were considered at high risk of bias, both for the studies included for primary outcome analysis (14/19; 73.7%) and for studies included for disorder-specific scales (16/21; 76.2%). The remaining studies were at low (3/19, 15.8%; 3/21, 14.3%, respectively) or intermediate risk of bias (2/19, 10.5%; 2/21, 9.5%, respectively). Contrary to what was initially planned in the registered protocol, no sensitivity analysis was conducted in the meta-analysis, because of the insufficient number of studies demonstrating a low risk of bias.

Limitations in blinding, especially neurostimulation blinding (10/19 and 11/21, respectively), and attrition bias (9/19 and 11/21, respectively), accounted for almost the totality of the high risk of bias. Conversely, outcome blinding or selective reporting of results were generally considered at low risk of bias. In most cases, methods of random sequence generation and randomization concealment were not reported. Only six of the 27 studies applied and reported appropriate randomization (48, 50, 52, 57, 63, 66). Overall, according to the GRADE guidelines (39), evidence from this study was considered of moderate quality.

## Discussion

The treatment of pathological anxiety in ADs, PTSD and MDD is faced with high levels of resistance. Focal brain stimulation is a promising therapeutic avenue. However, there is currently a lack of synthesis of results from neuromodulatory interventions for anxiety across those disorders. This review and meta-analysis summarized available evidence regarding the efficacy of focal brain stimulation on pathological anxiety, with a dimensional perspective. Results are globally in favor of a positive effect of neurostimulation at the end of interventions across ADs, PTSD and MDD, with a medium effect size, and a moderate quality of evidence. Active neuromodulation protocols also resulted in greater response and/or remission rates than control conditions. These results suggest that focal brain stimulation may be an interesting option for the treatment of pathological anxiety. However, long-term efficacy data were insufficient to conduct a meta-analytic study, being scarce and heterogeneous. Nevertheless, follow-up data were qualitatively in favor of a prolonged favorable effect of neurostimulation.

The meta-analysis purposely gathered results from heterogeneous conditions and protocols, which were investigated in subgroup analyses. Regarding stimulation techniques, both TMS and tDCS showed a tendency toward an improvement of anxiety symptom intensity and resulted in greater response and/or remission rates than control conditions immediately after treatment. Mild adverse events typical of the stimulation techniques were reported. DBS showed a tendency toward an improvement of anxiety symptom intensity immediately after treatment. However, the meta-analysis was limited to two studies on MDD, which included patients with moderate baseline anxiety levels. This analysis is not sufficient to determine the therapeutic potential of DBS. As a result, at present, only TMS and tDCS appear as potential neuromodulatory options for the treatment of pathological anxiety.

As for categorically defined disorders, positive effects were found in PTSD and GAD subgroups, on the basis of psychometric scores measuring either generic anxiety or disorder-specific scores. Positive effects were also found for MDD-related anxiety. In contrast, there was no evidence for positive impact of brain stimulation in PD, and the only trial on patients with SP showed no positive effects. No study was found for SAD. Overall, our results indicate that in categorial terms, PTSD and GAD may benefit from non-invasive brain stimulation.

Notably, ADs, PTSD and MDD were pooled in the main analysis, as a consequence of the dimensional approach of the study. This strategy is in line with the National Institute of Mental Health research domain criteria initiative, which organizes mental disorders in constructs, e.g., the negative valence system, and allows a multi-level integration of neuroscientific findings to identify biological targets for treatment (69). The choice of the disorders included was guided by a line of arguments pointing toward similarities in neurobiological bases. From a phenomenological perspective, they are internalizing disorders that share the highest levels of comorbidities and a great number of reciprocal risk interactions (5). In agreement with this, their susceptibility profiles and potential genetic risk factors also overlap (5). Moreover, brain activation patterns identified in ADs, MDD and PTSD are similar, implicating a fronto-limbic network (18, 21). On the contrary, obsessive-compulsive disorder was not included, as it is known to follow distinct, cortico–striato–thalamo–cortical pathogenic mechanisms (70). Likewise, somatic and dissociative disorders (1) were considered too different in their expression and pathophysiology, and were not included. Interestingly, the finding of an association between anxious et depressive symptoms reduction in the meta-regression analysis may further suggest the existence of common biological underpinnings, or, at least, of close interactions between the two phenomena.

Regarding the relative efficacy of brain stimulation depending on target brain regions, both right dlPFC inhibition and excitation tended to alleviate anxiety symptoms, with a larger effect size for right dlPFC activation. Left dlPFC activation had no global effect. These findings are overall consistent with the negative correlation identified between pathological anxiety levels and the resting state connectivity strength between right dlPFC and amygdala (71). However, other authors reported similar correlations, but with left dlPFC (72, 73). It should be emphasized that the classification of the net effect (excitatory vs. inhibitory) of brain stimulation is derived from simple observations of motor cortex responses, that may not directly translate to PFC. Moreover, the net effect of brain stimulation is dependent on a number of factors, including stimulation parameters (duration, intensity etc.), variability in subjects' intrinsic cortical excitability, and ongoing pharmacological treatments (74). Thus, the “excitatory” or “inhibitory” classification should be interpreted with caution. Future preclinical studies should specifically investigate this issue in the PFC. Moreover, it should be reminded that our analysis pooled various stimulation techniques and protocols. Therapeutic effects of focal brain stimulation also depend on the accurate placement of stimulation devices above targeted brain regions. In most studies included in the meta-analysis, target area was determined by scalp distances anterior to regions where motor responses were obtained. This placement method likely resulted in imprecise, heterogenous targeting. Indeed, it has been demonstrated that results obtained with such positioning methods differ from those obtained with gold standard, brain imaging-based stimulation device positioning (75, 76). Future clinical trials should aim to optimize stimulation parameters, and may primarily investigate the right dlPFC as a target, as our results would suggest.

Results of the present study are generally in agreement with, and extend results from previous reviews of the literature, including narrative reviews (31, 32, 34) and meta-analyses (35–37, 77, 78). Among narrative reviews, the most closely related to our work systematically reviewed the effectiveness of non-invasive brain stimulation for the treatment of anxiety disorders across RCTs and open-label studies (34). Interestingly, no clear evidence was reported in this work for a positive effect of brain stimulation in SP, on a larger number of trials than reported in our meta-analysis [including open-label studies; (34)]. This suggests that current neuromodulation strategies have no efficacy in SP. Contrary to our results, Vicario et al. (34) suggested that neuromodulation may improve PD, and that left dlPFC stimulation is more efficacious. However, the majority of studies included in the review were not sham-controlled, possibly accounting for those differences with our results.

Three meta-analyses were recently conducted for non-invasive brain stimulation in PTSD (36), rTMS in GAD (37), and rTMS in ADs (35), reaching similar conclusions to the present review. Evaluating the efficacy of non-invasive brain stimulation in PTSD across RCTs revealed a strong positive therapeutic effect of TMS (36). Cui et al. reported a positive effect for rTMS on GAD, based on a much larger study sample size than reported here (*n* = 21 vs. 3). This discrepancy is due to the inclusion of 19/21 studies published in Chinese language by Cui et al. (37). Most consisted in inhibitory stimulation of the right dlPFC. Cirillo et al. reported a reduction in clinical severity after TMS, both in GAD and in PTSD, using disorder-specific scales, and suggested that right dlPFC excitatory stimulation may be superior to targeting left dlPFC (35). Despite reaching conclusions consistent with those obtained in our meta-analysis, these studies were restricted to single diagnostic categories, and/or stimulation techniques, and/or disorder-specific instruments.

Prior to our work, another transdiagnosis meta-analysis had been published. Trevizol and colleagues conducted a meta-analysis investigating the effects of TMS on generic anxiety scores across ADs, PTSD and OCD, and found no evidence for a positive effect, including PD, PTSD and OCD subgroups (77). Most RCTs (8/14) reported interventions on OCD, possibly accounting for differences between conclusions from this and our work. Four of those negative OCD studies targeted the dlPFC, further supporting the distinct pathophysiology of OCD, and its exclusion from neurobiologically-based dimensional studies of anxiety. Differences for the PTSD subgroup may be partly accounted for by the publication after 2016 of studies included in our review. During the conduct of our study -i.e., after the protocol was pre-registered- a closely related work has been released by Vergallito et al. (78). Using both disorder-specific and generic anxiety scores, they found that rTMS and tDCS reduced anxiety levels across ADs. This main finding is in agreement with our primary and secondary analyses. Depression scores were also lower in intervention vs. control groups (78). In addition to our analysis, one study was included for which we could not obtain original results despite contacting the authors (79). In contrast with Vergallito et al., we ranked risk of bias as high in a majority of studies, whenever, the sham stimulation induced sounds and/or bodily sensations that might have been readily identified by intervention administrators or subjects. Our risk of bias classification is conservative, attributing high risk of bias if intervention administrators were not blinded, or if participants could guess the intervention group. For instance, we conservatively ranked 90° TMS coil placement as a high risk of performance bias. This classification may be mitigated by a meta-analysis reporting that blinding assessment was satisfactory in MDD participants receiving sham TMS with either sham coils or angled coil placement (80). However, in one study included here, while angled TMS placement was used and blinding quality was measured, patients guessed better than chance their allocation group (46). Notably, Vergallito et al. restricted the analysis to ADs as defined in DSM-5. As demonstrated by changes between the 4th and 5th versions of the manual, diagnostic categories are a moving target, and an obstacle for neurobiologically-informed research. Instead, similarities in the anxiety phenomenon across ADs, PTSD and MDD call for a dimensional evaluation, which constitutes the originality and relevance of the present study.

## Limitations

First despite performing an extensive search of literature databases and applying PRISMA recommendations, a limited number of studies were included, with relatively small sample sizes [except for two studies measuring disorder-specific symptoms; (63, 66)]. Second, the primary outcome of the meta-analysis (generic anxiety scales results) was the explicit primary outcome in only 5/19 of the studies included. Third, therapeutic efficacy was evaluated at the end of brain modulation trials, while long-lasting reduction of symptoms intensity is warranted. Fourth, as pharmacological treatments were often administered concomitantly with brain stimulation, the relative contribution of the two strategies and their interactions remain to be clarified. Fifth, although only RCTs were analyzed, the risk of bias was high in a majority of studies, including the three studies showing the strongest positive effects (42, 48, 55). This was mostly due to randomization methods (insufficiently described), attrition, and insufficient blinding. Moreover, we could not entirely rule out that publication bias had occurred.

## Future Perspectives

Larger-sized, multicentered studies with pre-specified primary outcomes are warranted to fully assess the potential efficacy of TMS, tDCS and DBS in pathological anxiety. Trials designed to demonstrate long-lasting therapeutic responses and remissions will be critical in order to support the application of brain stimulation strategies in daily practice. In this respect, the adoption of standard follow-up intervals will tremendously help reproducibility and generalizability of future findings. The relative contributions and interactions between medication, psychotherapy and neuromodulation will need to be clarified. In a dimensional view, generic anxiety scales should be used to assess outcomes, and inclusion criteria might even be defined in a dimensional manner. Care should also be taken to apply and report adequate randomization and blinding procedures, including measurements of blinding assessment. Moreover, sham conditions will need to be hardly distinguishable from active brain stimulation (81, 82).

Alternative brain targets should be investigated. It is likely that targeting dlPFC to treat pathological anxiety has been empirically carried forward -at least in part- from usual clinical practice in MDD [as indicated by (47)], and not necessarily informed by neuroscientific findings. Indeed, there is some, but limited evidence for the implication of dlPFC in pathological anxiety (71–73) and in the preclinical model of fear conditioning (83). In contrast, the anxiety circuit comprises key brain regions such as the medial PFC, the amygdala, and the insula (10, 84) as demonstrated by a recent meta-analysis of fMRI data (18). Their neuromodulation might provide greater therapeutic outcomes than targeting dlPFC. However, those key nodes are located deeply within the brain. Their modulation will require the application of invasive or novel non-invasive DBS techniques [e.g., H-coil TMS: e.g., (62, 85); interference-based deep tDCS: (86); transcranial ultrasounds: (87)].

Finally, several research lines aim to develop brain stimulation procedures in a precision medicine framework. In the spatial domain, neuromodulated regions could be targeted selectively in different patient “clusters” distinguished by symptoms and neurobiological (e.g., imaging) dimensions [e.g., (88)]. In the temporal domain, instead of delivering continuous patterns of regular pulses, stimulation paradigms could adapt to ongoing, pathological brain activities to restore physiological functioning [(i.e., “closed-loop” stimulation; e.g., (89)]. Overall, it is likely that empirical brain modulation will move toward precise, neurobiological data-based techniques.

## Conclusion

Current evidence suggests that focal brain stimulation may be beneficial to alleviate pathological anxiety across ADs, PTSD and MDD. Additional, large-scale, RCTs measuring long-term effects of neuromodulation on anxiety are warranted. Future research will expand and refine our knowledge in the perspective of offering precision medicine neuromodulation tools to manage pathological anxiety in the context of high treatment resistance levels.

## Data Availability Statement

The original contributions presented in the study are included in the article/Supplementary Material. Further inquiries can be directed to the corresponding author/s.

## Author Contributions

FG, FS, BA, and TB: study design. FG and TB: data collection and manuscript drafting. FG, FS, AS, and TB: data analysis. FG, FS, AS, BA, and TB: revisions and approval. All authors contributed to the article and approved the submitted version.

## Funding

This work was funded by a CCA Inserm Bettencourt grant to Thomas Bienvenu (R20005GS).

## Conflict of Interest

Unrelated to brain stimulation techniques, BA received speaker's honoraria and/or a travel allowance from Lundbeck, Sanofi, Janssen-Cilag, and Eli Lilly. He has served on the advisory board of Janssen-Cilag. The remaining authors declare that the research was conducted in the absence of any commercial or financial relationships that could be construed as a potential conflict of interest.

## Publisher's Note

All claims expressed in this article are solely those of the authors and do not necessarily represent those of their affiliated organizations, or those of the publisher, the editors and the reviewers. Any product that may be evaluated in this article, or claim that may be made by its manufacturer, is not guaranteed or endorsed by the publisher.
