# Supplementary material for: Neuromodulation Treatments of Pathological Anxiety in Anxiety Disorders, Stressor-Related Disorders, and Major Depressive Disorder: A Dimensional Systematic Review and Meta-Analysis
Source: Front Psychiatry. 2022 Jul 1;13:910897. doi: 10.3389/fpsyt.2022.910897 (PMC9283719; doi:10.3389/fpsyt.2022.910897)
Supplement: Supplementary file 1 [file Data_Sheet_1.docx]

**SUPPLEMENTARY MATERIAL**

**Studies selection**

*Literature search logics*

For the literature search, the following terms were used : ("brain stimulation" OR "TMS" OR "RTMS" OR "Transcranial Magnetic Stimulation" OR "Theta burst" OR "transcranial direct current stimulation" OR "TDCS" OR "DBS" OR "neuromodulation") AND ("Anxiety disorders" OR "agoraphobia" OR "social, Anxiety" OR "phobic disorders" OR "Panic disorder" OR "Phobia" OR "stress disorder, post-traumatic" OR "PTSD" OR "anxious symptoms" OR "HAMA" OR "Hamilton anxiety" OR "Anxiety scale" OR "Beck Anxiety Inventory")

*Additional studies potentially eligible, with missing data*

In the case of potentially eligible studies with missing information, authors were contacted via email(where possible, lead *and* last authors). A list of the studies for which we could not obtain data is provided below with details of 1) data of interest, and, 2) exchanges with study authors.

Full text articles, missing numerical values

1. Berman et al., 2000 (Berman et al., 2000)

- Outcomes included HARS, and HDRS for which we attempted to retrieve anxiety-somatization data.

- The lead author, Dr Robert Berman, no longer had access to the data.

1. Boggio et al., 2010 (Boggio et al., 2010)

- Outcomes included PCL and HARS

- No response was received to our emails.

1. Fizgerald et al., 2012 (Fitzgerald et al., 2012)

- Psychometric measurements included STAI and HDRS for which we attempted to retrieve anxiety-somatization data.

- No response was received to our emails.

1. Lee et al., 2019 (Lee et al., 2019)

- Outcomes included HARS, and HDRS for which we attempted to retrieve anxiety-somatization data.

- No response was received to our emails.

1. Nam et al., 2013 (Nam et al., 2013)

- Outcomes included CAPS

- No response was received to our emails.

1. Notzon et al., 2015 (Notzon et al., 2015)

- Outcomes included specific phobia questionnaire SPQ which we tried to retrieve for secondary outcome meta-analysis

- No response was received to our emails.

1. Rosenberg et al. 2002 (Rosenberg et al., 2002)

- Outcomes included HDRS for which we attempted to retrieve anxiety-somatization data.

- The lead author, Dr Paul M. Rosenberg, no longer had access to the data.

Studies published as abstracts

1. Armas-Castañeda et al., 2015 (Armas-Castañeda et al., 2015)

- Outcomes included HDRS (for which we attempted to retrieve anxiety-somatization data) and “anxiety scales”

- No response was received to our emails.

1. Danish Ahmed et al., 2016 (Danish Ahmed, 2016)

- Outcomes included HARS and HDRS, for which we attempted to retrieve anxiety-somatization data

- No response was received to our emails.

1. Huang et al., 2014 (Huang et al., 2014)

- Outcomes included HARS and HDRS, for which we attempted to retrieve anxiety-somatization data

- No response was received for this study.

1. Lin et al., 2019 (Lin et al., 2019)

- Outcomes included HARS and HDRS, for which we attempted to retrieve anxiety-somatization data

- No response was received for this study.

**Data extraction: particular cases**

One article was included which publication date is posterior to November 2020 (Smits et al., 2021). This article was then available in a pre-print version.

Extracted scales that were reported in the meta-analysis are reported in the main text (Table 1). Two studies measured both HARS and BAI scales (PO: (de Lima et al., 2019; Praško et al., 2007). We chose to extract HARS scores for generic anxiety score analysis because this scale was most consistently used in the studies. Likewise, PTSD CheckList (PCL) scale was extracted for meta-analysis from 5 trials reporting both PCL and Clinician Administered PTSD Scale for DSM-5 (CAPS-5) (PO: (Cohen et al., 2004; Leong et al., 2020; Watts et al., 2012); SO: (Kozel et al., 2018; Philip et al., 2019a)). Finally, one trial reported two sub scores of Acrophobia Questionnaire, one fore Anxiety and one for Avoidance. In the absence of total score, we chose to extract anxiety scores for the analysis as this dimension was most consistent with the aims of our study.

Two trials included population with anxiety disorder and comorbid MDD: one for GAD (Nasiri et al., 2020), and one for PD (Mantovani et al., 2013). These studies contained the corresponding anxiety disorders as sole (Nasiri et al., 2020) or joint primary diagnosis (Mantovani et al., 2013), and were accordingly classified in the corresponding anxiety disorders subgroups.

**Detailed reporting of included studies**

*Populations*

Raw group results of psychometric measures are presented in the main text **Table 3**. Characteristics of included studies are presented in the main text (Table 1) and in **Table 4.**

Mean age of participants and gender ratio have been described in the main text. The studies included 589 participants for the primary outcome (315 subjects in active arms and 279 in control arms; population sizes 5-85 subjects, median 30), and 712 subjects for the secondary outcome (377 in treatment arm, 347 in control arm; population sizes 9-103 subjects, median 29). As some studies were conducted in a cross-over design, the total number of participants is not equal to the sum of subjects in the active and control groups.

Thereafter, we refer to the studies included for generic anxiety scales (primary outcome) as “PO”, and to the studies included only for disorder-specific scales (secondary outcome) as “SO”. Studies included both in primary and secondary outcomes are described in primary outcome only.

Irrespective of the primary or secondary outcome, gender ratio was rather heterogeneous between studies and diagnoses, but notably, some PTSD trials included up to 100% males (SO: (Ahmadizadeh and Rezaei, 2018; van ’t Wout-Frank et al., 2019), probably due to particular recruitment in the military setting. Three trials did not report gender ratio (PO: (Puigdemont et al., 2015; Sadeghi Movahed et al., 2018), SO: (Kozel et al., 2018).

*Design of studies*

Conditions :

From all trials, most involved a parallel group comparison between active and sham stimulations (PO: (Ahmadizadeh et al., 2019; Diefenbach et al., 2016; Dilkov et al., 2017; Herrmann et al., 2017; Holtzheimer et al., 2017; Huang et al., 2018; de Lima et al., 2019; Mantovani et al., 2013; Praško et al., 2007; Watts et al., 2012), SO: (Anderson et al., 2007; Kozel et al., 2018; Philip et al., 2019b, 2019a; Smits et al., 2021; Triggs et al., 2010; van ’t Wout-Frank et al., 2019). Five studies included a third additional condition to this classical design: 3 with a second TMS protocol (PO: (Cohen et al., 2004; Leong et al., 2020); SO: (Ahmadizadeh and Rezaei, 2018)), one with pharmacotherapy, in comparison with tDCS (PO: (Sadeghi Movahed et al., 2018)), and one with exposure therapy as add-on to TMS (SO: (Isserles et al., 2013)). Only two trials did not involve a sham stimulation. Nasiri et al. compared tDCS in GAD to two control conditions consisting respectively of psychotherapy alone and waiting list (PO: (Nasiri et al., 2020)). Dastjerdi et al compared tDCS in MDD to cranial electrotherapy stimulation (CES) alone or associated with tDCS (Dastjerdi et al., 2015). CES is a noninvasive therapeutic device that applies pulsed, alternating microcurrent (<1000 μA) transcutaneously to the head. One trial included a healthy control group in addition to active and sham TMS stimulation conditions, (PO: (Deppermann et al., 2017)). Data from this group were not included in analysis. Two last trials employed a cross-over design, with consecutive subjects alternately receiving active or sham as the initial experimental condition (PO: (Osuch et al., 2009; Puigdemont et al., 2015)).

Most trials allowed continuation of pharmaco- or psychotherapy if there was no change in treatment a few weeks before and during the trial.

Blinding :

Two trials had a single blind design (PO: (Sadeghi Movahed et al., 2018); SO: (van ’t Wout-Frank et al., 2019)). Other trials had a double-blind design, but limitations in the protocol or in technical equipment impaired blinding quality, especially for administrators. In TMS studies, only two trials established real double-blind conditions, with specific sham coil identical to the active one, selected for each participant by independent technicians (PO: (Diefenbach et al., 2016); SO: (Philip et al., 2019a)). One of these two studies verified subject blinding quality with a dedicated questionnaire SO: ((Philip et al., 2019a)). In tDCS studies, lack of information concerning administrator blinding and sham device functioning was most frequent. In two studies, current was ramped down after a few seconds, but administrators had to turn off the system manually (PO: (de Lima et al., 2019; Ahmadizadeh et al., 2019) . In one study, the administrator had to enter a code related to the active or sham stimulation program, impeding adequate blinding .

Analysis scheme :

Out of the 27 RCTs analyzed, 7 studies conducted an intent to treat analysis (ITT) (PO: (Huang et al., 2018; de Lima et al., 2019; Mantovani et al., 2013; Praško et al., 2007; Puigdemont et al., 2015; Watts et al., 2012); SO: (Philip et al., 2019a, 2019b)); 14 studies a per protocol (PP) (PO: (Ahmadizadeh et al., 2019; Anderson et al., 2007; Cohen et al., 2004; Dilkov et al., 2017; Herrmann et al., 2017; Holtzheimer et al., 2017; Nasiri et al., 2020); SO: ((Isserles et al., 2013; Osuch et al., 2009; Smits et al., 2021)) or “modified ITT” analysis (PO: (Diefenbach et al., 2016; Leong et al., 2020); SO: (Ahmadizadeh and Rezaei, 2018; Kozel et al., 2018)). Of note, modified ITT analyses were considered in quality assessment as displaying a high risk of bias (see **Supplementary Table 1** below). Five studies did not report dropout rates, so their analysis types were classified as unclear (PO: (Dastjerdi et al., 2015; Deppermann et al., 2017; Sadeghi Movahed et al., 2018; Triggs et al., 2010); SO: (van ’t Wout-Frank et al., 2019)).

*Brain modulation protocols*

In TMS studies, the most frequent protocol used was 1 Hz targeting right dlPFC, evaluated in 8 studies (PO: (Cohen et al., 2004; Diefenbach et al., 2016; Leong et al., 2020; Mantovani et al., 2013; Praško et al., 2007; Watts et al., 2012); SO: (Kozel et al., 2018; Osuch et al., 2009)). Eight trials assessed high frequency stimulation of dlPFC. Three of the high frequency protocols were conducted on the left side (PO: (Anderson et al., 2007; Deppermann et al., 2017; Triggs et al., 2010): one at 10 Hz (Anderson et al., 2007), one at 5 Hz (Triggs et al., 2010), and one with iTBS protocol (Deppeerman et al., 2017). Six of high frequency protocols were conducted on on the right side: one at 5 Hz (PO: (Triggs et al., 2010)), two at 10 Hz (PO: (Cohen et al., 2004; Leong et al., 2020), two at 20 Hz (PO: (Dilkov et al., 2017), SO: (Ahmadizadeh and Rezaei, 2018)), and one with an intermittent Theta Burst Stimulation protocol (SO: ((Philip et al., 2019a)).

Two studies assessed medial PFC stimulation, at 10 Hz (PO: (Herrmann et al., 2017) and at 20 Hz with deep-TMS (SO: (Isserles et al., 2013)). Three trials tested the effect of other protocols than unilateral or medial PFC, using respectively bilateral dlPFC stimulation at 20 Hz (SO: (Ahmadizadeh and Rezaei, 2018)), posterior parietal cortex at 1 Hz (PO: (Huang et al., 2018)), and TMS synchronized to intrinsic alpha frequency (target not specified) (SO: (Philip et al., 2019b).

Transcranial magnetic stimulation protocols used various stimulation intensities, from 80% to 120% of motor threshold (MT). Similarly, there was a broad variability in within-session duration, from 3.5 to 40 minutes, and in total number of stimulation sessions, ranging from 10 to 30 sessions. Total pulse numbers ranged from 1,000 to 90,000, and were similar between high- and low-frequency stimulations protocols.

When specified, most trials used an 8-shape coil device (PO: (Anderson et al., 2007; Deppermann et al., 2017; Dilkov et al., 2017; Huang et al., 2018; Leong et al., 2020; Mantovani et al., 2013; Praško et al., 2007; Triggs et al., 2010; Watts et al., 2012), SO: (Ahmadizadeh and Rezaei, 2018; Kozel et al., 2018; Osuch et al., 2009), except two studies using circular coils (PO: (Cohen et al., 2004), SO: (Herrmann et al., 2017)) and one study using an H-coil (SO: (Isserles et al., 2013)). The types of coils used by Philip and colleagues for rTMS and sTMS were not specified (SO: (Philip et al., 2019b, 2019a)).

Two strategies were used for sham stimulation: first a sham coil supposed to look and sound identical to the active coil (SO: (Anderson et al., 2007; Diefenbach et al., 2016; Huang et al., 2018; Leong et al., 2020; Mantovani et al., 2013; Triggs et al., 2010; Watts et al., 2012); SO: (Ahmadizadeh and Rezaei, 2018; Herrmann et al., 2017; Isserles et al., 2013; Kozel et al., 2018; Philip et al., 2019b, 2019a)), and, second, the deviation of magnetic stimulation from cortical structures by placing the coil with a 90° angle from the scalp (PO: (Cohen et al., 2004; Deppermann et al., 2017; Dilkov et al., 2017; Praško et al., 2007); SO: (Osuch et al., 2009)). Only one study included a sham coil that sounded, looked and felt like the active condition (PO: (Leong et al., 2020)).

Stimulation coil positioning was mostly determined by distance from the motor strip location that caused hand movement (between 4 and 6 cm to target dlPFC: PO: (Anderson et al., 2007; Cohen et al., 2004; Dilkov et al., 2017; Leong et al., 2020; Mantovani et al., 2013; Praško et al., 2007; Triggs et al., 2010; Watts et al., 2012); SO: (Ahmadizadeh and Rezaei, 2018; Osuch et al., 2009)). Only one study employed MNI coordinates associated with neuronavigation to target the right dlPFC (PO: (Diefenbach et al., 2016)). Other studies used the 10–20 EEG system to target cortical regions underlying P4 (PO: (Huang et al., 2018)), F3 (PO: (Deppermann et al., 2017)), F4 electrode (SO: (Kozel et al., 2018; Philip et al., 2019a), PFz (SO: (Herrmann et al., 2017)) or did not specify the target location method (SO: (Philip et al., 2019b)).

In tDCS studies, 4 protocols targeted dlPFC. Two conducted cathodal stimulation of the right dlPFC (anode on the contralateral deltoid) (PO: (Nasiri et al., 2020; Sadeghi Movahed et al., 2018), one anodal stimulation of the left dlPFC (cathode on the right supraorbital area) (PO: (de Lima et al., 2019)), and one bilateral dlPFC stimulation (anode left, cathode right) (PO: (Ahmadizadeh et al., 2019)). Three trials targeted other brain areas: one with cathodal stimulation of the inferior fusiform gyrus (SO: (Smits et al., 2021)), one with anodal stimulation of vmPFC (SO: (van ’t Wout-Frank et al., 2019)), and one setting targets based on EEG patterns (without precisions; PO: (Dastjerdi et al., 2015)).

Stimulation parameters were consistent across studies: current intensity was always set at 2 mA, (except for one trial: 1,25 mA; SO: (Smits et al., 2021)), and stimulations lasted for 5 to 10 sessions of 20 to 30 minutes.

For sham conditions, two studies used a current ramp-down after 30 seconds (PO: (Ahmadizadeh et al., 2019; de Lima et al., 2019)), and the last study proceeded to 6 seconds fade-in fade-out stimulation at the start and end of the stimulation period, interleaved by occasional 15 ms pulses of 0.11 mA (SO: (Smits et al., 2021)). Three studies did not report how sham stimulation were established (PO: (Dastjerdi et al., 2015; Sadeghi Movahed et al., 2018); SO: (van ’t Wout-Frank et al., 2019)).

When electrode positioning methods were reported, the 10–20 EEG system was consistently used (PO: (Ahmadizadeh et al., 2019; de Lima et al., 2019; Sadeghi Movahed et al., 2018) ; SO : (Smits et al., 2021; van ’t Wout-Frank et al., 2019)).

In the two DBS trials bilateral subcallosal cingulate DBS was assessed in MDD (PO: (Holtzheimer et al., 2017; Puigdemont et al., 2015)). In Holtzheimer et al., stimulation lasted for 6 months, at 130 Hz, 91 µs pulse width, and 4 mA intensity, progressively increased to 8 mA in case of partial response. An additional 6- month open label phase was conducted after blinded period. In Puigdemont et al., subjects were stimulated for 3 months, at 130-135 Hz, 120-240 µs pulse width, and 3.5-5 V electrical voltage. They were then followed until loss to follow-up.

*Adverse events*

Side effects for each study are reported in the main texte **table 3.**

In TMS studies, ten serious adverse events were described among 15 studies : three cases of generalized tonic-clonic seizures (one during the 20^th^ session of active rTMS; PO: (Dilkov et al., 2017)), one during the 8^th^ session of active deepTMS (SO: (Isserles et al., 2013)), and one four days after last active treatment (PO: (Anderson et al., 2007)), one suicidal ideation emergence in active group (PO: (Leong et al., 2020)), one homicidal ideation emergence and one hospitalization for suicidality during follow-up (both receiving sham stimulation) (SO: (Philip et al., 2019a)), two manic and one hypomanic episodes (PO: (Anderson et al., 2007; Cohen et al., 2004)), and one chest pain occurrence (in active group, considered to be unrelated to the intervention; PO: (Diefenbach et al., 2016)). Numerous mild side effects were reported, including headaches and pain at the stimulation point, were the most frequently reported side effects (PO: (Anderson et al., 2007; Cohen et al., 2004; Diefenbach et al., 2016; Herrmann et al., 2017; Huang et al., 2018; Mantovani et al., 2013; Triggs et al., 2010); SO: (Ahmadizadeh and Rezaei, 2018; Isserles et al., 2013; Kozel et al., 2018; Philip et al., 2019b, 2019a)), followed by neck pain (PO: (Cohen et al., 2004; Herrmann et al., 2017; Huang et al., 2018; Mantovani et al., 2013; Triggs et al., 2010)), facial pain (PO: (Cohen et al., 2004; Diefenbach et al., 2016; Triggs et al., 2010)) and transient dizziness (PO: (Diefenbach et al., 2016; Herrmann et al., 2017; Triggs et al., 2010); SO: (Dilkov et al., 2017)). When specified, there was no statistically significant difference between active and sham condition for side effect rates, except for facial twitch in one study (PO: (Diefenbach et al., 2016)). Only one study reported impaired concentration for 2 subjects in the active group (PO: (Triggs et al., 2010)). One study reported two cases of ear discomfort (PO: (Cohen et al., 2004)), one reported a case of nausea in active group (SO: (Philip et al., 2019a)). Finally, one trial did not register any serious or mild side effect (PO: (Praško et al., 2007)).

Three studies did not report side effects (PO: (Deppermann et al., 2017; Watts et al., 2012); SO: (Osuch et al., 2009).

Concerning tDCS studies, three did not report on side effects (PO: (Nasiri et al., 2020; Sadeghi Movahed et al., 2018); SO: (van ’t Wout-Frank et al., 2019)). No severe adverse event were described in four other studies. Mild effects occurrence was reported in three studies (PO: (Ahmadizadeh et al., 2019; de Lima et al., 2019); SO: (Smits et al., 2021)), with mainly headaches, tingling, sleepiness, mild itching or burning sensations on the scalp and light skin redness in the second one (SO: (Smits et al., 2021)). Only sleepiness, sensations on the scalp and skin redness were significantly more frequent in active groups. Finally, no serious or mild side effect was found in one study (PO: (Dastjerdi et al., 2015)).

DBS : detailed side effects have been previously described in the main text for Holtzheimer et al. They were collected by Puigdemont et al., but not described in the article.

*Risk of bias analysis*

In Supplementary Table 1 we report details regarding the reasons for risk of bias ranking for each study and each item.

| **Study reference** | **Random sequence generation (selection bias)** | **Allocation concealment (selection bias)** | **Blinding of participants and personnel (performance bias)** | **Blinding of outcome assessment (detection bias)** | **Incomplete outcome data (attrition bias)** | **Selective reporting (reporting bias)** | **Other bias** | **Overall risk of bias for study (score and category)** |
| --- | --- | --- | --- | --- | --- | --- | --- | --- |
| Anderson et al., 2007 (PO) | Randomization stratified by degree of treatment resistance. | Sealed envelope. | Subjects blind, administrator blinding NS. Of 25 participants, 76% guessed their correct treatment allocation. | NS | Per protocol analysis (4 dropouts not included). | No indication of selective reporting. | No indication of other bias. | **High: 2**  **Intermediate: 2**  **Low: 3** |
| Ahmadizadeh et al., 2019  (PO+SO) | Randomly assigned by an independent statistician. | NS | Double blind design.  Efficacy of Patients' blinding verified.  Administrators reported as blind but their intervention to turn off DCS is described. | Raters blind. | Per protocol (6 dropouts excluded from analysis). | No indication of selective reporting. | No indication of other bias. | **High: 1**  **Intermediate: 3**  **Low: 3** |
| Cohen et al., 2004  (PO+SO) | NS | NS | Double blind design.  Patients “blind” but coil held differently between sham and active groups + different frequencies. Administrator unblinded. | Raters blind (therapist). | Per protocol (5 dropouts excluded from analysis). | No indication of selective reporting. | No indication of other bias. | **High: 2**  **Intermediate: 2**  **Low: 3** |
| Dastjerdi et al., 2015  (PO) | No precise information.  Baseline imbalance in anxiety severity (p=0.02). | NS | Double blind design.  No information about administrators blinding. | NS | NS | No indication of selective reporting. | No indication of other bias. | **High: 1**  **Intermediate: 4**  **Low: 2** |
| De Lima et al., 2019  (PO+SO) | Computerized random number generation. | NS | Double blind design but no specification. | Raters blind. | No missing data or dropouts. | No indication of selective reporting. | No indication of other bias. | **High: 0**  **Intermediate: 2**  **Low: 5** |
| Deppermann et al., 2017  (PO+SO) | NS | NS | Double blind design.  Patients guessed better than chance their allocation. No information regarding administration blinding. | Raters blind. | Potential attrition (indication of last observation carried forward in case of missing questionnaire). | No indication of selective reporting. | No indication of other bias. | **High: 2**  **Intermediate: 2**  **Low: 3** |
| Diefenbach et al., 2016  (PO+SO) | Computerized random number generation and  randomization and schedule created to replace for attrition. | NS | Double blind design.  Subjects blind. Administrator blind (setting of the apparatus by independent technician). | Raters blind (clinical psychologist). | ITT (1 patient excluded after randomization from active group, 8 withdrew before randomization). Imputation to replace missing data. | No indication of selective reporting. | No indication of other bias. | **High: 0**  **Intermediate: 1**  **Low: 6** |
| Dilkov et al., 2017  (PO) | Treatment randomization table generated by a statistician, placed in sealed envelopes, received by individual RTMS performer. | Sealed envelopes | Double blind design.  Different positioning of the apparatus in the 2 groups: administrator unblinded. | Raters blind. | 10 patients from active group non included in analysis (5 before treatment, 5 after treatment because stimulation protocol was not correctly applied). | No indication of selective reporting. | No indication of other bias. | **High: 2**  **Intermediate: 0**  **Low: 5** |
| Hermann et al., 2017 (PO+SO) | Simple randomization 1-1, controlling for the factor sex. No information on sequence generation.  No baseline imbalance on "final sample" n=39. | No information. | Participants + exposure therapists blind. No information about admonistrators blinding.  The number of participants who believed to have received active stimulation did not differ between the sham and active. | Raters blind. | Missing data.  Per Protocol analysis. | A lot of analysis. | No indication of other bias. | **High: 1**  **Intermediate: 4**  **Low: 2** |
| Holtzheimer et al., 2017 (PO) | Randomisation computer generated (SAS version 9.2) with a block size of three at each site before the site started the study. | NS | Double blind design. At each site, an unblinded DBS programmer was informed of treatment allocation; all other team members and patients were masked to treatment allocation. | Raters blind. | Per protocol analysis (n=90, n=85 included in analysis for primary outcome). | No indication of selective reporting. | No indication of other bias. | **High: 1**  **Intermediate: 2**  **Low: 4** |

**Supplementary table 1: Risk of bias assessment (1/3).**

| **Study reference** | **Random sequence generation (selection bias)** | **Allocation concealment (selection bias)** | **Blinding of participants and personnel (performance bias)** | **Blinding of outcome assessment (detection bias)** | **Incomplete outcome data (attrition bias)** | **Selective reporting (reporting bias)** | **Other bias** | **Overall risk of bias for study (score and category)** |
| --- | --- | --- | --- | --- | --- | --- | --- | --- |
| Huang et al., 2018  (PO+SO) | NS | NS | Double blind design.  Subjects blind. No information about administrators blinding. | Raters blind. | No missing data or dropouts. | No indication of selective reporting. | No indication of other bias. | **High: 0**  **Intermediate: 3**  **Low: 4** |
| Leong et al., 2020  (PO+SO) | Random sequence generation. | Allocation concealment by the envelope method. | Double blind design.  Subjects blind. Administrator unblinded. | Raters blind (psychiatrists). | Modified ITT (n=31 randomized, n=29 included in "ITT analysis"). | No indication of selective reporting. | No indication of other bias. | **High: 2**  **Intermediate: 0**  **Low: 5** |
| Mantovani et al., 2013  (PO+SO) | NS | NS | Double blind design. Subjects assessment of blinding not different between groups. Administrator unmasked but isolated. | Raters blind. | No missing data or dropouts. | No indication of selective reporting. | No correction for multiple comparison. | **High: 1**  **Intermediate: 3**  **Low: 3** |
| Movahed et al., 2018  (PO+SO) | NS | NS | Single blinding (subjects). | NS | NS | No indication of selective reporting. | No indication of other bias. | **High: 1**  **Intermediate: 4**  **Low: 2** |
| Nasiri et al., 2020  (PO+SO) | Roll of a dice. | NS | Double blind design.  Subjects received easily distinguishable interventions. Administrators blind (therapist unaware of group assignment; TDCS provider unaware of study design). | Raters blind. | Per protocol (4 dropouts excluded from analysis). | No indication of selective reporting. | No indication of other bias. | **High: 3**  **Intermediate: 1**  **Low: 3** |
| Prasko et al., 2007  (PO+SO) | NS | NS | Subjects blind but two different coil positions, administrators unblinded. | Raters blind. | No missing data or dropouts. | No indication of selective reporting. | No indication of other bias. | **High: 1**  **Intermediate: 2**  **Low: 4** |
| Puigdemont et al., 2015 (PO) | Random allocation sequence generated using the pseudorandom numbers generator in SPSS software version 18, no blocking was used. | Sealed envelope selection used to conceal the sequence. | Only the investigator who manipulated the neurostimulator knew the allocation pertaining to each individual. | Clinical assessments performed by a single psychiatrist, blinded to the stimulation assignment. | LOCF method. ITT analyse. | No indication of selective reporting. | No indication of other bias. | **High: 0**  **Intermediate: 0**  **Low: 7** |
| Triggs et al., 2020 (PO) | Randomization 1:1:1 then in sham group randomized 1:1 between left and right. No information on sequence generation. | NS | Double blind design. Subjects blind (not tested), administrators unblinded. | Raters blind. | 13 withdrewed consent, not clear if before or after randomization. No information about dropouts. Unknown analysis. | No indication of selective reporting. | No indication of other bias. | **High: 1**  **Intermediate: 3**  **Low: 3** |
| Watts et al., 2012  (PO+SO) | NS | NS | Double blind design but no specification. | Raters blind (psychologists). | No missing data or dropouts. | No indication of selective reporting. | No indication of other bias. | **High: 0**  **Intermediate: 3**  **Low: 4** |
| **Primary outcome studies (n=17)** | High: 2  Intermediate: 10  Low: 5 | High: 0  Intermediate: 14  Low: 3 | High: 10  Intermediate: 6  Low: 1 | High: 0  Intermediate: 3  Low: 14 | High: 8  Intermediate: 3  Low: 6 | High: 0  Intermediate: 0  Low: 17 | High: 0  Intermediate: 1  Low: 16 | **High: 20**  **Intermediate: 36**  **Low: 63** |

**Supplementary table 1: Risk of bias assessment (2/3).**

| **Study reference** | **Random sequence generation (selection bias)** | **Allocation concealment (selection bias)** | **Blinding of participants and personnel (performance bias)** | **Blinding of outcome assessment (detection bias)** | **Incomplete outcome data (attrition bias)** | **Selective reporting (reporting bias)** | **Other bias** | **Overall risk of bias for study (score and category)** |
| --- | --- | --- | --- | --- | --- | --- | --- | --- |
| Ahmadizadeh et al., 2018  (SO) | NS |  | Double blind design.  Subjects blind. Administrators unblinded. | Raters blind. | Modified ITT: 7 dropouts. | No indication of selective reporting. | No indication of other bias. | **High: 2**  **Intermediate: 2**  **Low: 3** |
| Isserles et al., 2013  (SO) | NS | NS | Double blind design.  Subjects blind. No information about administrators blinding. | Raters blind. | Modified ITT: 5 dropouts. | No indication of selective reporting. | No indication of other bias. | **High: 1**  **Intermediate: 3**  **Low: 3** |
| Kozel et al., 2018  (SO) | Computer generated randomization. | Sealed envelopes. | Double blind design  Subjects blind. RTMS performer unmasked but isolated from other staff members. | Raters blind. | Modified ITT.  1/103 baseline data excluded from analysis. | No indication of selective reporting. | No indication of other bias. | **High: 2 Intermediate: 0**  **Low: 5** |
| Osuch et al., 2009 | NS | NS | Subjects blind (not tested) but two different coil positions, administrators not blind. | Raters blind. | One dropout not included in sham period. per protocol analysis | No indication of selective reporting. | No indication of other bias. | **High: 2**  **Intermediate: 2**  **Low: 3** |
| Philip, Barredo et al., 2019  (SO) | Randomization was performed by a study member un- involved with TBS delivery, in a 1:1 design stratified by symptom severity and sex. | NS | Double blind.  Subjects assessment of blinding not different between groups. Administrator blind (unaware of coil model active/sham). | Raters blind. | ITT (imputation to replace missing data, or maximum likelihood parameter estimation in mixed models). | No indication of selective reporting. | No indication of other bias. | **High: 0**  **Intermediate: 2**  **Low: 5** |
| Philips, Aiken et al., 2019 bis (sTMS) | Randomization in blocks of four, balanced across both sides. No information about randomization sequence generation. | NS | Subjects blind (sham coil). Administrators blinding not reported. Participants were not able to accurately guess their group assignment. | Raters blinding NS. | ITT analysis. | No indication of selective reporting. | No indication of other bias. | **High: 0**  **Intermediate: 4**  **Low: 2** |
| Smits et al., 2021  (SO) | Randomization with a stimulator activating code from a randomized list, stratified by psychotherapy. | Experimenters and subjects blind for code-to-condition correspondence | Double blind.  Subjects blind.  Administrators blind. | Autoevaluation only. | Per protocol analysis: 3 dropouts (primary outcome) / 4 dropouts (secondary analysis of PCL scale). | Pre-registered protocol. | No indication of other bias. | **High: 1**  **Intermediate: 0**  **Low: 6** |
| Van ’t Wout-Frank et al., 2019  (SO) | NS | NS | Single blind (subjects). | NS | NS | No indication of selective reporting. | No indication of other bias. | **High: 1**  **Intermediate: 4**  **Low: 2** |
| **Secondary outcome (n=20)** | High : 1  Intermediate : 15  Low : 5 | High : 0  Intermediate : 18  Low : 3 | High : 11  Intermediate : 7  Low : 3 | High : 0  Intermediate : 3  Low : 18 | High : 11  Intermediate : 2  Low : 8 | High : 0  Intermediate : 1  Low : 20 | High : 0  Intermediate : 1  Low : 20 | **High : 23**  **Intermediate : 47**  **Low : 77** |

**Supplementary table 1: Risk of bias assessment (3/3).**

PO: primary outcome (anxiety symptoms intensity); SO: secondary outcome (disorder-specific scales)

**References**

Ahmadizadeh, M.-J., and Rezaei, M. (2018). Unilateral right and bilateral dorsolateral prefrontal cortex transcranial magnetic stimulation in treatment post-traumatic stress disorder: A randomized controlled study. Brain Research Bulletin *140*, 334–340.

Ahmadizadeh, M.J., Rezaei, M., and Fitzgerald, P.B. (2019). Transcranial direct current stimulation (tDCS) for post-traumatic stress disorder (PTSD): A randomized, double-blinded, controlled trial. Brain Research Bulletin *153*, 273–278.

Anderson, I.M., Delvai, N.A., Ashim, B., Ashim, S., Lewin, C., Singh, V., Sturman, D., and Strickland, P.L. (2007). Adjunctive fast repetitive transcranial magnetic stimulation in depression. Br J Psychiatry *190*, 533–534.

Armas-Castañeda, G., Ricardo-Garcell, J., Romo-Nava, F., Heinze-Martin, G., and González-Olvera, J.J. (2015). Transcranial Magnetic Stimulation: antidepressant efficacy and three-month follow-up. Brain Stimulation *8*, 343.

Berman, R.M., Narasimhan, M., Sanacora, G., Miano, A.P., Hoffman, R.E., Hu, X.S., Charney, D.S., and Boutros, N.N. (2000). A randomized clinical trial of repetitive transcranial magnetic stimulation in the treatment of major depression. Biological Psychiatry *47*, 332–337.

Boggio, P.S., Rocha, M., Oliveira, M.O., Fecteau, S., Cohen, R.B., Campanhã, C., Ferreira-Santos, E., Meleiro, A., Corchs, F., Zaghi, S., et al. (2010). Noninvasive Brain Stimulation With High-Frequency and Low-Intensity Repetitive Transcranial Magnetic Stimulation Treatment for Posttraumatic Stress Disorder. J. Clin. Psychiatry *71*, 992–999.

Cohen, H., Kaplan, Z., Kotler, M., Kouperman, I., Moisa, R., and Grisaru, N. (2004). Repetitive Transcranial Magnetic Stimulation of the Right Dorsolateral Prefrontal Cortex in Posttraumatic Stress Disorder: A Double-Blind, Placebo-Controlled Study. AJP *161*, 515–524.

Danish Ahmed, S. (2016). A Randomized Sham-Controlled Study of Transcranial Direct Current Stimulation in Depression. Indian Journal of Psychiatry *58*.

Dastjerdi, G., Mirhoseini, H., and Mohammadi, E. (2015). Investigating the Synergistic Effects of Transcranial Direct Current Stimulation and Cranial Electrical Stimulation in Treatment of Major Depression in A Double Blinded Controlled Trial. Biomed. Pharmacol. J *8*, 1267–1274.

Deppermann, S., Vennewald, N., Diemer, J., Sickinger, S., Haeussinger, F.B., Dresler, T., Notzon, S., Laeger, I., Arolt, V., Ehlis, A.-C., et al. (2017). Neurobiological and clinical effects of fNIRS-controlled rTMS in patients with panic disorder/agoraphobia during cognitive-behavioural therapy. NeuroImage: Clinical *16*, 668–677.

Diefenbach, G.J., Bragdon, L.B., Zertuche, L., Hyatt, C.J., Hallion, L.S., Tolin, D.F., Goethe, J.W., and Assaf, M. (2016). Repetitive transcranial magnetic stimulation for generalised anxiety disorder: A pilot randomised, double-blind, sham-controlled trial. Br J Psychiatry *209*, 222–228.

Dilkov, D., Hawken, E.R., Kaludiev, E., and Milev, R. (2017). Repetitive transcranial magnetic stimulation of the right dorsal lateral prefrontal cortex in the treatment of generalized anxiety disorder: A randomized, double-blind sham controlled clinical trial. Progress in Neuro-Psychopharmacology and Biological Psychiatry *78*, 61–65.

Fitzgerald, P.B., Hoy, K.E., Herring, S.E., McQueen, S., Peachey, A.V.J., Segrave, R.A., Maller, J., Hall, P., and Daskalakis, Z.J. (2012). A double blind randomized trial of unilateral left and bilateral prefrontal cortex transcranial magnetic stimulation in treatment resistant major depression. Journal of Affective Disorders *139*, 193–198.

Herrmann, M.J., Katzorke, A., Busch, Y., Gromer, D., Polak, T., Pauli, P., and Deckert, J. (2017). Medial prefrontal cortex stimulation accelerates therapy response of exposure therapy in acrophobia. Brain Stimulation *10*, 291–297.

Holtzheimer, P.E., Husain, M.M., Lisanby, S.H., Taylor, S.F., Whitworth, L.A., McClintock, S., Slavin, K.V., Berman, J., McKhann, G.M., Patil, P.G., et al. (2017). Subcallosal cingulate deep brain stimulation for treatment-resistant depression: a multisite, randomised, sham-controlled trial. The Lancet Psychiatry *4*, 839–849.

Huang, Z., Li, Y., Zhan, S., Li, N., Ding, Y., Hou, Y., Wang, L., and Wang, Y. (2014). P1060: Suppressing cortical hyperexcitability of right parietal cortex by repetitive transcranial magnetic stimulation (rTMS) relieved the anxiety symptom of patients with generalized anxiety disorder. Clinical Neurophysiology *125*, S278.

Huang, Z., Li, Y., Bianchi, M.T., Zhan, S., Jiang, F., Li, N., Ding, Y., Hou, Y., Wang, L., Ouyang, Q., et al. (2018). Repetitive transcranial magnetic stimulation of the right parietal cortex for comorbid generalized anxiety disorder and insomnia: A randomized, double-blind, sham-controlled pilot study. Brain Stimulation *11*, 1103–1109.

Isserles, M., Shalev, A.Y., Roth, Y., Peri, T., Kutz, I., Zlotnick, E., and Zangen, A. (2013). Effectiveness of Deep Transcranial Magnetic Stimulation Combined with a Brief Exposure Procedure in Post-Traumatic Stress Disorder – A Pilot Study. Brain Stimulation *6*, 377–383.

Kozel, F.A., Motes, M.A., Didehbani, N., DeLaRosa, B., Bass, C., Schraufnagel, C.D., Jones, P., Morgan, C.R., Spence, J.S., Kraut, M.A., et al. (2018). Repetitive TMS to augment cognitive processing therapy in combat veterans of recent conflicts with PTSD: A randomized clinical trial. Journal of Affective Disorders *229*, 506–514.

Lee, S., Jang, K.-I., Yoon, S., and Chae, J.-H. (2019). The Efficacy of Miniaturized Repetitive Transcranial Magnetic Stimulation in Patients with Depression. Clin Psychopharmacol Neurosci *17*, 409–414.

Leong, K., Chan, P., Ong, L., Zwicker, A., Willan, S., Lam, R.W., and McGirr, A. (2020). A Randomized Sham-controlled Trial of 1-Hz and 10-Hz Repetitive Transcranial Magnetic Stimulation (rTMS) of the Right Dorsolateral Prefrontal Cortex in Civilian Post-traumatic Stress Disorder: Un essai randomisé contrôlé simulé de stimulation magnétique transcrânienne repetitive (SMTr) de 1 Hz et 10 Hz du cortex préfrontal dorsolatéral droit dans le trouble de stress post-traumatique chez des civils. Can J Psychiatry *65*, 770–778.

de Lima, A.L., Braga, F.M.A., da Costa, R.M.M., Gomes, E.P., Brunoni, A.R., and Pegado, R. (2019). Transcranial direct current stimulation for the treatment of generalized anxiety disorder: A randomized clinical trial. Journal of Affective Disorders *259*, 31–37.

Lin, Y., Zhang, C., and Wang, Y. (2019). A Randomized Controlled Study of Transcranial Direct Current Stimulation in Treatment of Generalized Anxiety Disorder. Brain Stimulation *12*, 403.

Mantovani, A., Aly, M., Dagan, Y., Allart, A., and Lisanby, S.H. (2013). Randomized sham controlled trial of repetitive transcranial magnetic stimulation to the dorsolateral prefrontal cortex for the treatment of panic disorder with comorbid major depression. Journal of Affective Disorders *144*, 153–159.

Nam, D.-H., Pae, C.-U., and Chae, J.-H. (2013). Low-frequency, Repetitive Transcranial Magnetic Stimulation for the Treatment of Patients with Posttraumatic Stress Disorder: a Double-blind, Sham-controlled Study. Clin Psychopharmacol Neurosci *11*, 96–102.

Nasiri, F., Mashhadi, A., Bigdeli, I., Chamanabad, A.G., and Ellard, K.K. (2020). Augmenting the unified protocol for transdiagnostic treatment of emotional disorders with transcranial direct current stimulation in individuals with generalized anxiety disorder and comorbid depression: A randomized controlled trial. Journal of Affective Disorders *262*, 405–413.

Notzon, S., Deppermann, S., Fallgatter, A., Diemer, J., Kroczek, A., Domschke, K., Zwanzger, P., and Ehlis, A.-C. (2015). Psychophysiological effects of an iTBS modulated virtual reality challenge including participants with spider phobia. Biological Psychology *112*, 66–76.

Osuch, E.A., Benson, B.E., Luckenbaugh, D.A., Geraci, M., Post, R.M., and McCann, U. (2009). Repetitive TMS combined with exposure therapy for PTSD: A preliminary study. Journal of Anxiety Disorders *23*, 54–59.

Philip, N.S., Barredo, J., Aiken, E., Larson, V., Jones, R.N., Shea, M.T., Greenberg, B.D., and van ’t Wout-Frank, M. (2019a). Theta-Burst Transcranial Magnetic Stimulation for Posttraumatic Stress Disorder. AJP *176*, 939–948.

Philip, N.S., Aiken, E.E., Kelley, M.E., Burch, W., Waterman, L., and Holtzheimer, P.E. (2019b). Synchronized transcranial magnetic stimulation for posttraumatic stress disorder and comorbid major depression. Brain Stimulation *12*, 1335–1337.

Praško, J., Záleský, R., Bareš, M., Horáček, J., Kopeček, M., Novák, T., and Pašková, B. (2007). The effect of repetitive transcranial magnetic stimulation (rTMS) add on serotonin reuptake inhibitors in patients with panic disorder: A randomized, double blind sham controlled study. 7.

Puigdemont, D., Portella, M., Pérez-Egea, R., Molet, J., Gironell, A., Diego-Adeliño, J. de, Martín, A., Rodríguez, R., Àlvarez, E., Artigas, F., et al. (2015). A randomized double-blind crossover trial of deep brain stimulation of the subcallosal cingulate gyrus in patients with treatment-resistant depression: a pilot study of relapse prevention. J Psychiatry Neurosci *40*, 224–231.

Rosenberg, P.B., Mehndiratta, R.B., Mehndiratta, Y.P., Wamer, A., Rosse, R.B., and Balish, M. (2002). Repetitive Transcranial Magnetic Stimulation Treatment of Comorbid Posttraumatic Stress Disorder and Major Depression. J Neuropsychiatry Clin Neurosci *14*, 270–276.

Sadeghi Movahed, F., Alizadeh Goradel, J., Pouresmali, A., and Mowlaie, M. (2018). Effectiveness of Transcranial Direct Current Stimulation on Worry, Anxiety, and Depression in Generalized Anxiety Disorder: A Randomized, Single-Blind Pharmacotherapy and Sham-Controlled Clinical Trial. Iran J Psychiatry Behav Sci *12*.

Smits, F.M., Geuze, E., Schutter, D.J.L.G., van Honk, J., and Gladwin, T.E. (2021). Effects of tDCS during inhibitory control training on performance and PTSD, aggression and anxiety symptoms: a randomized-controlled trial in a military sample. Psychol. Med. 1–11.

Triggs, W.J., Ricciuti, N., Ward, H.E., Cheng, J., Bowers, D., Goodman, W.K., Kluger, B.M., and Nadeau, S.E. (2010). Right and left dorsolateral pre-frontal rTMS treatment of refractory depression: A randomized, sham-controlled trial. Psychiatry Research *178*, 467–474.

Watts, B.V., Landon, B., Groft, A., and Young-Xu, Y. (2012). A sham controlled study of repetitive transcranial magnetic stimulation for posttraumatic stress disorder. Brain Stimulation *5*, 38–43.

van ’t Wout-Frank, M., Shea, M.T., Larson, V.C., Greenberg, B.D., and Philip, N.S. (2019). Combined transcranial direct current stimulation with virtual reality exposure for posttraumatic stress disorder: Feasibility and pilot results. Brain Stimulation *12*, 41–43.
